# Subtype-Specific Roles of Anterior Cingulate Cortex Neurons in Pain-Induced Social Deficits in Mice

**DOI:** 10.7150/thno.129482

**Published:** 2026-02-11

**Authors:** Xiangdong Wan, Ziqian Yan, Zhaoyichun Zhang, Xueqing Liu, Dingding Yang, Ming Zhang, Haiying Liu, Jiaqi Li, Bo Yang, Rong Zheng, Yifan Lu, Jing Huang, Fan Zhang, Guohong Cai, Shengxi Wu

**Affiliations:** 1 Department of Neurobiology, School of Basic Medicine, Fourth Military Medical University, Xi'an, China.; 2 The Key Laboratory of Neural and Vascular Biology, Ministry of Education and Department of Biochemistry and Molecular Biology, Hebei Medical University, Shijiazhuang, China.; 3 Graduate School of Hebei Medical University, Shijiazhuang, China.; 4 State Key Laboratory of Military Stomatology and National Clinical Research Center for Oral Diseases and Shaanxi Clinical Research Center for Oral Diseases, Department of Orthodontics, School of Stomatology, Fourth Military Medical University, Xi'an, China.; 5 Department of Nuclear Medicine, Xijing Hospital, Fourth Military Medical University, Xi'an, China.

**Keywords:** anterior cingulate cortex, GABAergic neurons, CaMKII⁺ neurons, chronic pain, social behavior

## Abstract

**Rationale:**

Pain is frequently accompanied by impairments in social behavior; however, the neural circuitry underlying pain-induced social deficits remains poorly understood. The aim of the present study was to delineate the distinct functional roles of γ-aminobutyric acid-releasing (GABAergic) neurons and calcium/calmodulin-dependent protein kinase II-positive (CaMKII^+^) neurons in the anterior cingulate cortex (ACC) in mediating pain-induced social deficits.

**Methods:**

Mouse models of inflammatory and neuropathic pain were employed. Optogenetic and chemogenetic approaches, combined with fiber photometry, were used to manipulate and monitor the activity of ACC neuronal subtypes. Social behaviors were assessed using the three-chamber social interaction test. Mechanical and thermal pain sensitivity were evaluated using von Frey filaments and the Hargreaves test, respectively.

**Results:**

Mice with chronic pain exhibited deficits in social preference and novelty. *In vivo* calcium imaging revealed that, during social interaction under pain conditions, the activity of ACC GABAergic neurons was reduced, whereas that of CaMKII^+^ neurons was increased. Chemogenetic manipulation demonstrated functional dissociation between these neuronal populations: activation of GABAergic neurons alleviated pain hypersensitivity but failed to rescue social deficits, whereas inhibition of these neurons improved pain-induced social deficits. Conversely, inhibition of CaMKII⁺ neurons attenuated hyperalgesia, while their activation partially restored social preference. Further analyses identified distinct interneuron subtype contributions, with parvalbumin-positive (PV^+^) neurons regulating both pain and pain-induced social preference deficits, and somatostatin-positive (SST⁺) neurons selectively mediating pain-induced social novelty deficits. These findings indicate that ACC neuronal subtypes exert complementary yet specialized roles in the comorbidity of pain and social deficits.

**Conclusions:**

Distinct ACC neuronal subtypes differentially regulate pain and social behaviors, revealing a functional “conflict” within the ACC whereby modulation of a single neuronal population cannot simultaneously ameliorate both pain and social deficits. These results underscore the necessity of circuit- and subtype-specific intervention strategies to disentangle and therapeutically target pain-related social deficit.

## Introduction

Pain is a complex phenomenon encompassing both sensory and emotional-discriminative components, extending beyond nociceptive processing to profoundly affect psychological and social well-being 1. Although extensive research has examined pain-associated comorbidities, including anxiety 2, depression 3, and cognitive impairment 4, comparatively little attention has been devoted to the impact of pain on social behavior. Chronic pain can disrupt social interactions and ultimately lead to social deficit, thereby promoting social isolation and diminishing quality of life 5. Social behavior forms the foundation of interpersonal relationships and social support networks, and impairments in social function are commonly manifested as social withdrawal, reduced social engagement, and declining social skills. These deficits not only exacerbate the psychological burden imposed by pain but may also establish a vicious cycle in which increasing isolation further aggravates pain-related outcomes. Despite its clear clinical relevance, the neural mechanisms underlying pain-induced social deficits remain poorly understood.

The anterior cingulate cortex (ACC) is a key brain region involved in both pain processing and social behavior. The ACC comprises diverse neuronal populations that exert distinct and sometimes opposing influences on these functions. Under chronic pain conditions, calcium/calmodulin-dependent protein kinase II-positive (CaMKII^+^) excitatory neurons in the ACC exhibit increased activity, a change that has been associated with pain memory formation and affective suffering 6. In contrast, the activity of γ-aminobutyric acid-releasing (GABAergic) neurons in the ACC is disrupted, with alterations in synaptic plasticity that may contribute to heightened pain sensitivity 7. GABAergic interneurons within the ACC are further subdivided into functionally heterogeneous subpopulations, most prominently parvalbumin-positive (PV^+^), somatostatin-positive (SST^+^), and vasoactive intestinal peptide-positive (VIP^+^) neurons 8. PV^+^ interneurons primarily regulate pyramidal neuron output and are essential for maintaining the excitation-inhibition balance and integrating sensory information. SST^+^ interneurons preferentially target distal dendrites of pyramidal neurons and modulate sensory input processing 9. VIP^+^ interneurons predominantly inhibit other inhibitory neurons, thereby forming disinhibitory microcircuits 10. Accumulating evidence suggests that these GABAergic subtypes are differentially affected in chronic pain states. For example, PV^+^ neurons have been implicated in pain modulation and the facilitation of hyperalgesia 11, and PV^+^ neurons in the ACC have also been linked to social deficits in mice 12. In contrast, dysfunction of SST^+^ neurons has been associated with pruritic behaviors 13. However, the precise contributions of distinct GABAergic interneuron subtypes within the ACC to pain-induced social deficits remain largely undefined.

Given the dual involvement of the ACC in pain processing and social behavior, we hypothesized that distinct neuronal populations—including excitatory neurons and specific subtypes of GABAergic interneurons—regulate pain perception and social behavior through separable mechanisms. We further proposed that social deficits arising during chronic pain are not merely secondary consequences of persistent nociception but instead reflect functional alterations within defined ACC neuronal subpopulations. Accordingly, this study sought to address two fundamental questions: first, how do different ACC neuronal subtypes regulate pain and social behavior under physiological conditions; and second, whether pain-induced social deficits are mediated by specific neuronal subsets within the ACC.

To address these questions, we employed the complete Freund's adjuvant (CFA) model of inflammatory pain and the spared nerve injury (SNI) model of neuropathic pain to examine the relationship between chronic pain and alterations in social behavior in mice. Optogenetic and chemogenetic approaches were used to selectively manipulate the activity of excitatory neurons, inhibitory interneurons, and defined ACC neuronal subtypes. Social behavioral changes were assessed during the early phase of chronic pain (2 days after CFA injection and 5 days after SNI). Finally, *in vivo* fiber photometry was used to monitor calcium dynamics in ACC neurons during social interactions following the establishment of chronic pain.

## Materials and Methods

### Animals

All experimental procedures were performed using 8-week-old adult mice. C57BL/6J and GAD67-green fluorescent protein (GAD67-GFP) mice were obtained from the Laboratory Animal Center of the Fourth Military Medical University. GAD2-Cre, PV-Cre, SST-Cre, VIP-Cre, and CaMKII-Cre transgenic mouse lines were purchased from Jiangsu Jinzhihe Biotechnology Co., Ltd. Animals were housed under standardized conditions (22-24 °C, 50% relative humidity) on a 12 h light/dark cycle (lights on from 08:00 to 20:00), with ad libitum access to food and water. All experimental protocols were approved by the Institutional Animal Care and Use Committee of the Fourth Military Medical University (approval number: 20211201) and were conducted in accordance with the National Institutes of Health guidelines for the care and use of laboratory animals.

### Pain models

#### Inflammatory pain model

Inflammatory pain was induced by intraplantar injection of complete Freund's adjuvant (CFA; Sigma-Aldrich) into the plantar surface of the right hind paw at volumes of 5, 10, or 20 μL. Control animals received equivalent volumes of sterile saline injected into the same anatomical site.

#### Neuropathic pain model

Neuropathic pain was induced using the SNI model. Briefly, a skin incision was made at the mid-thigh level of the right hindlimb. The biceps femoris muscle was bluntly dissected to expose the sciatic nerve and its three terminal branches. Distal to the trifurcation, the common peroneal and tibial nerves were tightly ligated and transected, followed by removal of approximately 2-4 mm of the distal nerve segments, while the sural nerve was left intact. In sham-operated mice, the sciatic nerve and its branches were exposed but left intact, and the muscle and skin were closed in layers using sutures.

### Three chamber social test

Social behavior was assessed using a three-chamber apparatus consisting of three interconnected compartments (40 × 20 cm each) and two wire-mesh cages (height: 17 cm). The test consisted of three consecutive phases: Habituation: The test mouse was placed in the central chamber and allowed to freely explore all compartments for 5 min. Social preference test: A age- and sex-matched stranger mouse was placed inside a wire-mesh cage in the left chamber, while an empty wire-mesh cage was placed in the right chamber. The test mouse was allowed to explore freely for 10 min, and sociability was assessed. Social novelty test: A second novel, age- and sex-matched unfamiliar mouse was placed in the wire-mesh cage in the right chamber, while the first mouse remained in the left chamber. Exploration behavior was recorded for an additional 10 min. Behavior was recorded using a video camera and analyzed using SMART 3.0 software.

### Pain threshold tests

#### Mechanical nociceptive threshold

Mechanical sensitivity was assessed using von Frey filaments. Mice were placed on an elevated wire-mesh platform and allowed to acclimate for approximately 40 min in individual enclosures. Calibrated von Frey filaments were applied perpendicularly to the mid-plantar surface of the right hind paw using the up-down method to determine the 50% paw withdrawal threshold.

#### Thermal nociceptive threshold

Thermal sensitivity was measured using a Hargreaves radiant heat apparatus. Baseline withdrawal latency was adjusted to 9-12 s, with a cutoff time of 25 s to prevent tissue damage. Three measurements were obtained per animal at 10 min intervals, and the mean withdrawal latency was calculated.

### Microdialysis

Mice were anesthetized with isoflurane (5.0% for induction and 2.0% for maintenance) and secured in a stereotaxic apparatus. A microdialysis guide cannula (CMA Microdialysis, Sweden) was stereotaxically implanted into the left ACC at the following coordinates: anterior-posterior (AP) +0.8 mm, medial-lateral (ML) ±0.3 mm, and dorsal-ventral (DV) -1.75 mm. After recovery, a concentric microdialysis probe was inserted, and artificial cerebrospinal fluid or saline was perfused at a flow rate of 1.5 μL/min for 1 h to allow equilibration. Microdialysis samples were collected at 20 min intervals before and 2 days after CFA injection. Samples were stored at -80 °C until analysis. Neurochemical analysis was performed using an ExionLC™ 20AC high-performance liquid chromatography (HPLC) system (AB Sciex, USA).

### Virus injection and fiber implantation

Mice were anesthetized by intraperitoneal injection of 1% pentobarbital sodium (60 mg/kg) and mounted in a stereotaxic apparatus (RWD Life Science Inc., China). Viral vectors (200 nL per site) were unilaterally infused into the ACC at the following stereotaxic coordinates relative to bregma: AP +0.8 mm, ML ±0.3 mm, and DV -1.75 mm. Injections were performed using a calibrated microinjection system consisting of a glass micropipette (Shanghai Gaoge Industry & Trade Co., Ltd., China) connected to a microprocessor-controlled microsyringe pump (KD Scientific Inc., USA), with an infusion rate of 35 nL/min. The viral vectors used included: rAAV-EF1α-DIO-hChR2(H134R)-mCherry (titer: 3.75 × 10¹² vg/mL; BrainVTA); rAAV-EF1α-DIO-eNpHR3.0-mCherry (titer: 2.18 × 10¹² vg/mL; BrainVTA); rAAV-EF1α-DIO-mCherry (titer: 3.12 × 10¹² vg/mL; BrainVTA); rAAV-EF1α-DIO-hM3D(Gq)-mCherry (titer: 3.03 × 10¹² vg/mL; BrainVTA); rAAV-EF1α-DIO-hM4D(Gi)-mCherry (titer: 2.43 × 10¹² vg/mL; BrainVTA); rAAV-hSyn-hM3D(Gq)-mCherry (titer: 5.35 × 10¹² vg/mL; BrainVTA); rAAV-hSyn-hM4D(Gi)-mCherry (titer: 5.09 × 10¹² vg/mL; BrainVTA); and rAAV-hSyn-DIO-GCaMP6s (titer: 2.2 × 10¹² vg/mL; injection volume: 200 nL into the left ACC; BrainVTA). For fiber photometry experiments, viral vectors were injected into the left ACC only, whereas optogenetic and chemogenetic manipulations involved bilateral injections. Seven days after viral delivery, a customized optical fiber (diameter of 200 μm Newdoon Inc., China) was stereotactically implanted 300 μm dorsal to the injection site. Animals were allowed a minimum postoperative recovery period of 14 days before behavioral testing.

### Intravenous viral injection

Mice were restrained in a holder designed for tail vein injection, and the tail was warmed using a heating lamp to facilitate vein visualization. rAAV-CaMKIIα-EGFP (titer: 5.06 × 10¹² vg/mL; diluted 1:2; BrainVTA) was slowly injected into the tail vein using a microsyringe at a total volume of 200 μL.

### Optical fiber photometry recording

Three weeks after viral transduction and fiber cannula implantation in the ACC, mice underwent behavioral testing. Excitation light power was adjusted to 0.01-0.02 mW, measured at the fiber tip. Calcium-dependent fluorescence signals were recorded using a fiber photometry system (Thinker Tech, Nanjing, China. Fluorescence signals were normalized as ΔF/F = (F - F₀)/F₀, where F represents the instantaneous fluorescence signal and F₀ denotes the median baseline fluorescence. To quantify calcium responses, the area under the curve (AUC) was calculated for the pre-stimulus (-2 to 0 s) and post-stimulus (0 to 6 s) periods.

### Chemogenetic manipulation

After intracranial delivery of chemogenetic viral vectors, mice were allowed a 4-week period for viral expression and recovery. Clozapine N-oxide (CNO; Tocris) was administered intraperitoneally from a stock solution of 1 mg/mL. Doses were adjusted by genotype: 1 mg/kg for GAD2-Cre, PV-Cre, SST-Cre, and CaMKII-Cre mice, and 5 mg/kg for VIP-Cre mice 10. Behavioral testing, including the three-chamber social assay and pain threshold measurements, was conducted approximately 40 min after CNO administration.

### Optogenetic manipulation

Following optogenetic viral injection, mice were allowed a 4-week period for viral expression and recovery. Animals were habituated to the experimental apparatus for 50 min before testing. Implanted fiber-optic patch cords were connected to a laser light source controlled by a waveform generator. Channelrhodopsin-2 (hChR2) was activated using 473 nm blue light (20 Hz, 40 ms pulse width, 5-7 mW output), whereas halorhodopsin (eNpHR)-mediated inhibition was achieved using 589 nm yellow light delivered continuously at 5-7 mW. Behavioral testing commenced immediately upon light delivery.

### *In vivo* optogenetic calcium imaging of GABAergic modulation of excitatory neurons in the ACC

To assess the influence of GABAergic neurons on excitatory neuronal activity in the ACC, rAAV-DIO-eNpHR-EGFP was injected into the ACC of 8-week-old male GAD2-Cre mice. One week later, rAAV-CaMKIIα-GCaMP6s-dTomato was injected into the same region, followed by implantation of an optical fiber cannula. After 21 days to allow for viral expression, optogenetic inhibition experiments were performed using 594 nm yellow light at intensities of 0, 5, and 15 mW/mm²). The stimulation paradigm consisted of 1 s light pulses followed by 9 s interstimulus intervals, repeated cyclically. Calcium activity in CaMKII⁺ neurons was monitored in real time using a fiber photometry system. Pre-stimulation (-1 to 0 s) and post-stimulation (0 to 4 s) periods were defined, and ΔF/F values were analyzed to calculate the AUC for quantitative comparison.

### Immunofluorescence

Mice were anesthetized by intraperitoneal injection of 1% pentobarbital sodium (60 µL/g) and transcardially perfused with 25 mL of ice-cold phosphate-buffered saline (PBS; 0.01 M, pH 7.4), followed by 50 mL of 4% paraformaldehyde (PFA) solution. Brains were post-fixed in 4% PFA for 2 h and subsequently cryoprotected in 30% sucrose solution for 36 h. Coronal sections containing the ACC were cut at a thickness of 35 µm using a freezing microtome (CM1950; Leica Microsystems, USA). Free-floating sections were blocked for 1 h at room temperature in Tris buffer (pH 7.4) containing 3% bovine serum albumin (BSA) and 0.3% Triton X-100. Sections were incubated overnight at 4 °C with the following primary antibodies: CaMKII (rabbit; GeneTex; Cat. No. GTX135117; 1:1000), GABA (rabbit; GeneTex; Cat. No. GTX125988; 1:400), c-Fos (rabbit; Synaptic Systems; Cat. No. 226008; 1:800), PV (rabbit; Swant; Cat. No. PV27; 1:500), SST (rabbit; Sigma-Aldrich; Cat. No. HPA019472; 1:400), VIP (rabbit; GeneTex; Cat. No. GTX129461; 1:300), and neuronal nuclei (NeuN; mouse; Abcam; Cat. No. ab104224; 1:500). Nuclear counterstaining was performed using 4′,6-diamidino-2-phenylindole (DAPI; Sigma-Aldrich; Cat. No. D9564; 1:800). After washing, sections were incubated for 2 h at room temperature with species-appropriate secondary antibodies conjugated to Alexa Fluor 488 or Alexa Fluor 594. Sections were then washed and mounted for imaging.

### Quantification and statistical analyses

Data analysis was performed using GraphPad Prism version 9.0 (GraphPad Software, USA), SPSS, and MATLAB R2018a (MathWorks, USA). All results are presented as mean ± standard of the mean (SEM). Statistical analyses included unpaired Student's *t* tests, paired Student's *t* tests, Mann-Whitney *U* tests, and Wilcoxon signed-rank tests, as appropriate. A *p* value < 0.05 was considered statistically significant.

## Results

### Pain induces social deficits in mice

**Figure 1:**
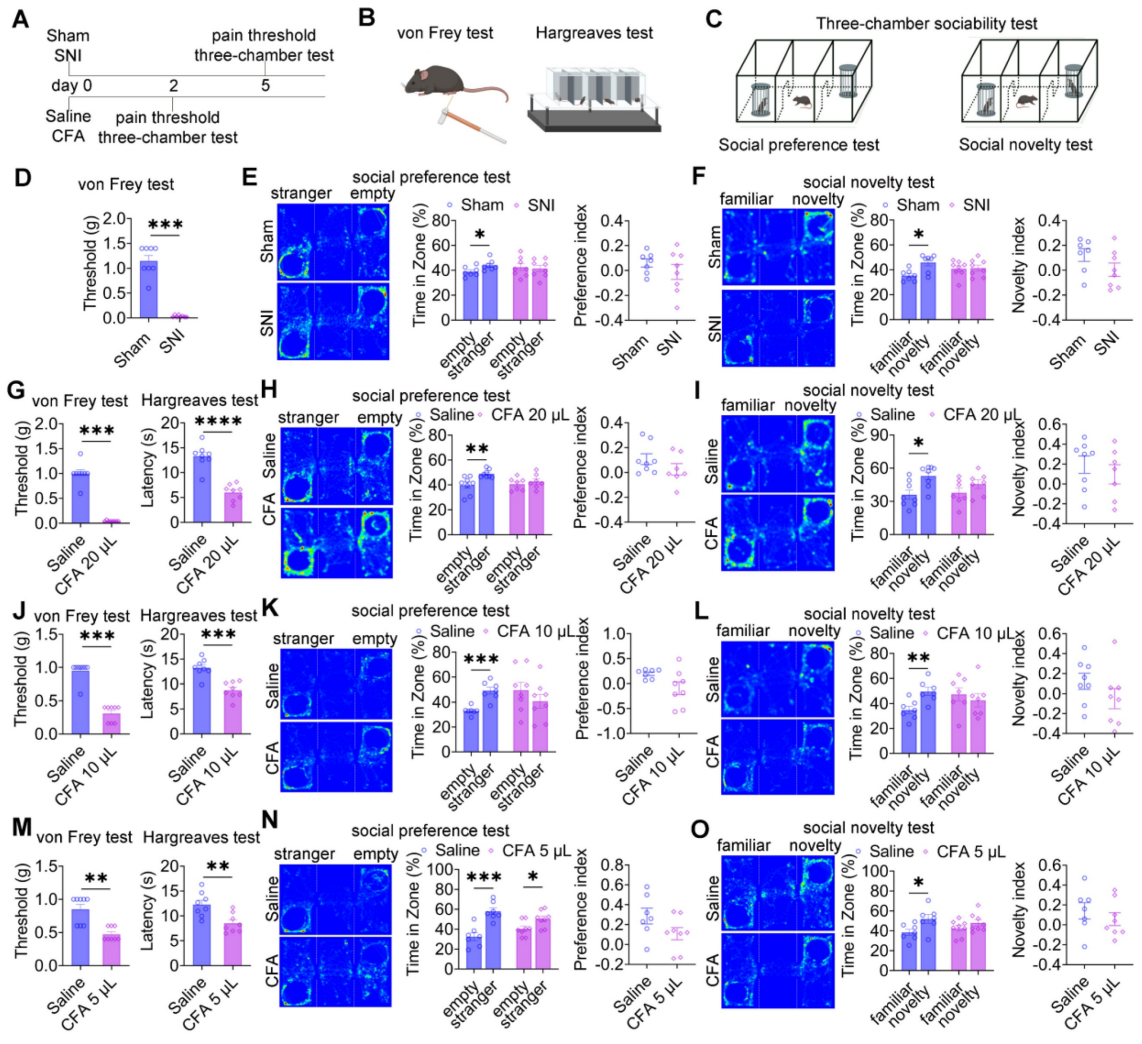
** Pain induces social deficits in mice.** (A) Experimental timeline. (B-C) Schematic illustrations of von Frey, Hargreaves, and three-chamber tests. (D) Mechanical withdrawal thresholds in sham and SNI mice (Sham: n = 8, SNI: n = 8). (E) Heatmaps and quantification of social interaction time and social preference index during the social preference test in sham and SNI mice (Sham: n = 7; SNI: n = 8). (F) Heatmaps and quantification of social interaction time and social novelty preference index in sham and SNI mice (Sham: n = 7, SNI: n = 8). (G) Mechanical and thermal pain thresholds following 20 μL CFA injection (Saline: n = 8, CFA: n = 8). (H-I) Heatmaps and quantification of social interaction time and preference indices during social preference and social novelty tests in saline and 20 μL CFA mice (Saline: n = 8, CFA: n = 7). (J) Mechanical and thermal pain thresholds following 10 μL CFA injection (Saline: n = 8, CFA: n = 8) (K-L) Heatmaps and quantification of social interaction time and preference indices during social preference and social novelty tests in saline and 10 μL CFA mice (Saline: n = 7, CFA: n = 7). (M) Mechanical and thermal pain thresholds following 5 μL CFA injection (Saline: n = 8, CFA: n = 8). (N-O) Heatmaps and quantification of social interaction time and preference indices during social preference and social novelty tests in saline and 5 μL CFA mice (Saline: n = 7; CFA: n = 8). Data are presented as mean ± SEM. Statistical tests and *n* values are indicated in the panels.

To examine the impact of pain on social behavior, two established pain models were employed: neuropathic pain induced by SNI and inflammatory pain induced by CFA injection (Figure 1A). Mechanical allodynia and thermal hyperalgesia were assessed using the von Frey filament test and the Hargreaves test, respectively (Figure 1B), while social behavior was evaluated using the three-chamber sociability test (Figure 1C). Five days after surgery, SNI mice exhibited significant mechanical allodynia compared with sham controls (Figure 1D). In the social preference test, sham mice displayed normal social preference, whereas SNI mice showed a marked reduction in social interaction time with the social stimulus (Figure 1E). Notably, the social preference index did not differ significantly between groups (Figure 1E). In the social novelty test, sham mice demonstrated a clear preference for the novel social stimulus, whereas SNI mice exhibited impaired social novelty behavior (Figure 1F), again without a significant difference in the preference index. Longitudinal analyses at 30 and 60 days post-SNI revealed persistent mechanical hypersensitivity (Figure S1A) and sustained deficits in both social preference and social novelty (Figures S1B-C), indicating that chronic neuropathic pain induces long-lasting social deficits. In the inflammatory pain model, intraplantar injection of 20 μL CFA induced significant thermal hyperalgesia by day 2 (Figure 1G). In the social preference test, saline-treated mice displayed normal social preference, whereas mice receiving 20 μL CFA exhibited reduced social interaction with the social stimulus (Figure 1H), despite preserved preference indices. Similarly, CFA-treated mice showed deficits in social novelty behavior (Figure 1I) with intact novelty preference indices. To examine dose-dependent effects, lower CFA volumes (10 μL and 5 μL) were administered. Both doses produced significant mechanical allodynia and thermal hyperalgesia within 2 days (Figures 1J and 1M). Mice treated with 10 μL CFA exhibited deficits in both social preference and social novelty (Figures 1K and 1L) while maintaining normal preference indices. In contrast, mice treated with 5 μL CFA retained normal social preference (Figure 1N) but displayed impaired social novelty behavior (Figure 1O), again without alterations in preference indices.

Analysis of locomotor activity revealed no significant differences in total distance traveled between sham and SNI mice during either the social preference and social novelty phases (Figures S2A-B), indicating that the observed social deficits were not attributable to impaired locomotion. In contrast, CFA-treated mice exhibited significant reduction in total distance traveled compared with saline controls (Figures S2C-D). Importantly, these findings dissociate locomotor suppression associated with inflammatory pain from the observed social behavioral deficits, which were evident across both inflammatory and neuropathic pain models.

### Alleviating pain ameliorates pain-induced social deficits in mice

**Figure 2:**
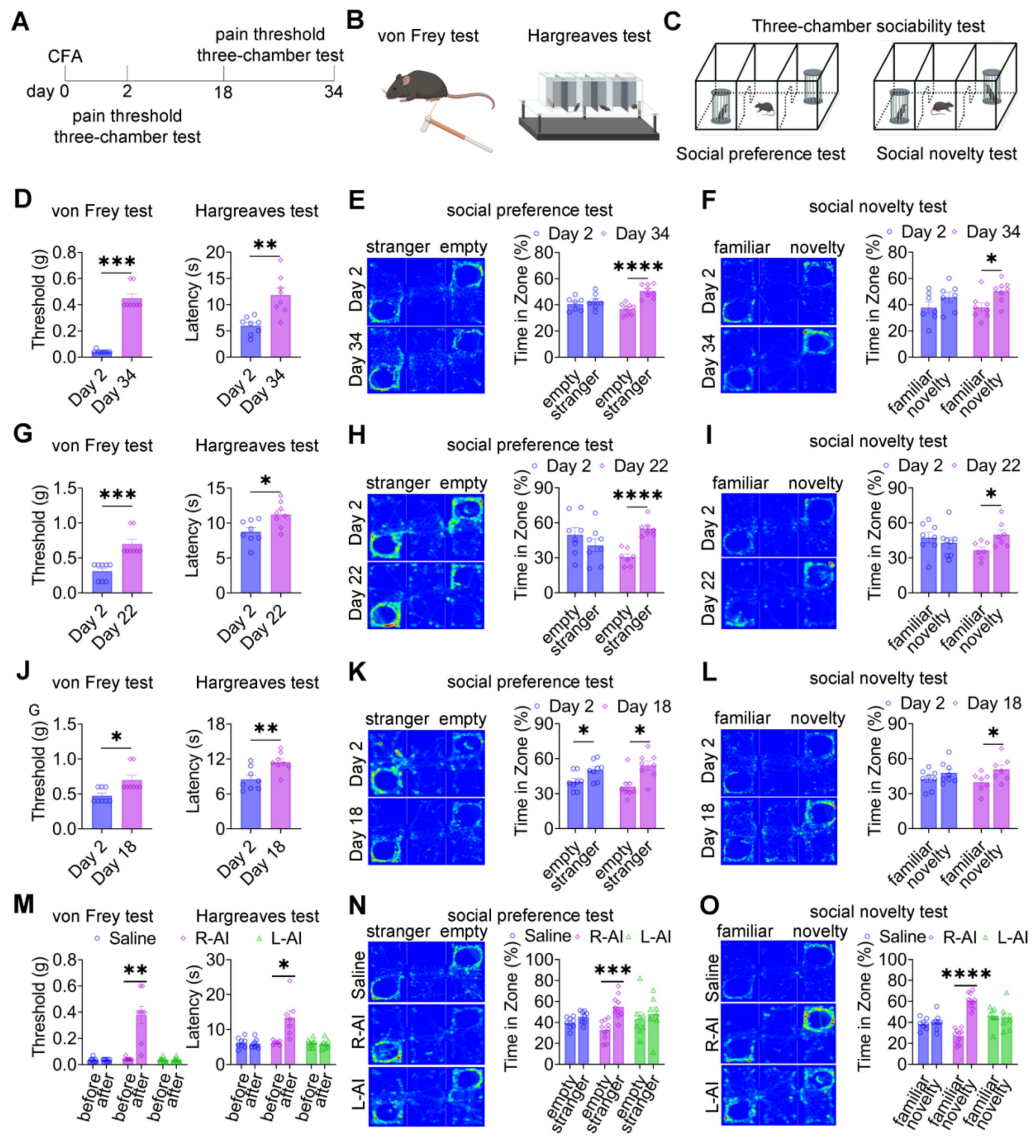
** Pain relief ameliorates pain-induced social deficits.** (A) Experimental timeline. (B-C) Schematics of von Frey, Hargreaves, and three-chamber tests. (D) Mechanical and thermal pain thresholds at day 2 and day 34 after 20 μL CFA injection (day 2: n = 8, day 34: n = 8). (E-F) Heatmaps and quantification of social interaction time during social preference and social novelty tests at day 2 and day 34 after 20 μL CFA injection (day 2: n = 7, day 34: n = 8). (G) Mechanical and thermal pain thresholds at day 2 and day 22 after 10 μL CFA injection (day 2: n = 8, day 22: n = 8). (H-I) Heatmaps and quantification of social interaction time during social preference and social novelty tests at day 2 and day 22 after 10 μL CFA injection (day 2: n = 8, day 22: n = 7). (J) Mechanical and thermal pain thresholds at day 2 and day 18 after 5 μL CFA injection (day 2: n = 8, day 18: n = 8). (K-L) Heatmaps and quantification of social interaction time during social preference and social novelty tests at day 2 and day 18 after 5 μL CFA injection (day 2: n = 8, day 18: n = 8). (M) Mechanical and thermal pain thresholds following saline, R-AICAR, or L-AICAR treatment (Saline: n = 8, R-AICAR: n = 8, L-AICAR, n = 8). (N-O) Heatmaps and quantification of social interaction time during social preference and social novelty tests following saline, L-AICAR and R-AICAR treatment (Saline: n = 8, R-AICAR: n = 10, L-AICAR: n = 8). Data are presented as mean ± SEM. Statistical tests and *n* values are indicated in the panels.

Given the close association between pain expression and social deficits, we next asked whether reducing pain severity could reverse pain-induced social impairments (Figure 2A-C). In mice injected with 20, 10, or 5 μL CFA, mechanical and thermal hypersensitivity gradually resolved, returning to baseline levels by day 34 (Figure 2D), day 22 (Figure 2G), and day 18 (Figure 2J), respectively. In parallel with the normalization of nociceptive thresholds, three-chamber testing revealed restoration of both social preference and social novelty behaviors in mice treated with 20 μL (Figures 2E-F), 10 μL (Figures 2H-I), and 5 μL (Figures 2K-L) CFA at the corresponding time points. These findings indicate that spontaneous resolution of inflammatory pain is accompanied by recovery of normal social behavior. To further examine whether targeted analgesia could directly rescue social deficits, the analgesic agent, 5-aminoimidazole-4-carboxamide ribonucleotide (AICAR), was locally administered into the CFA-injected hind paw 48 h after CFA challenge. Subcutaneous injection of AICAR into the ipsilateral hind paw (R-AICAR) significantly increased both mechanical and thermal pain thresholds within 2 h after injection (Figure 2M), confirming effective analgesia. Subsequent three-chamber testing demonstrated a restoration of social preference and social novelty behaviors compared with saline-treated controls (Figures 2N-O). In contrast, contralateral administration of AICAR into the left hind paw (L-AICAR) did not alter mechanical or thermal pain thresholds (Figure 2M), and social deficits persisted in both social preference and novelty tests (Figures 2N-O). These results indicate that AICAR-mediated rescue of social behavior depends on localized pain relief rather than centrally mediated effects.

To further probe the bidirectional relationship between pain and social behavior, SNI mice underwent passive socialization with female mice for 15 days after surgery (Figures S3A-C). Mechanical pain thresholds did not differ between passively socialized and control SNI mice (Figure S3D); however, passively socialized mice exhibited restored social novelty behavior (Figures S3E-F). Similar effects were observed in CFA-treated mice: passive socialization failed to alter mechanical or thermal nociceptive thresholds (Figure S3G) but normalized social preference behavior (Figures S3H-I). Together, these findings demonstrate that social interaction can partially ameliorate pain-induced social deficits without directly modifying nociceptive sensitivity.

### Pain activation of ACC CaMKII^+^ neurons alters excitatory/inhibitory neurotransmission

**Figure 3:**
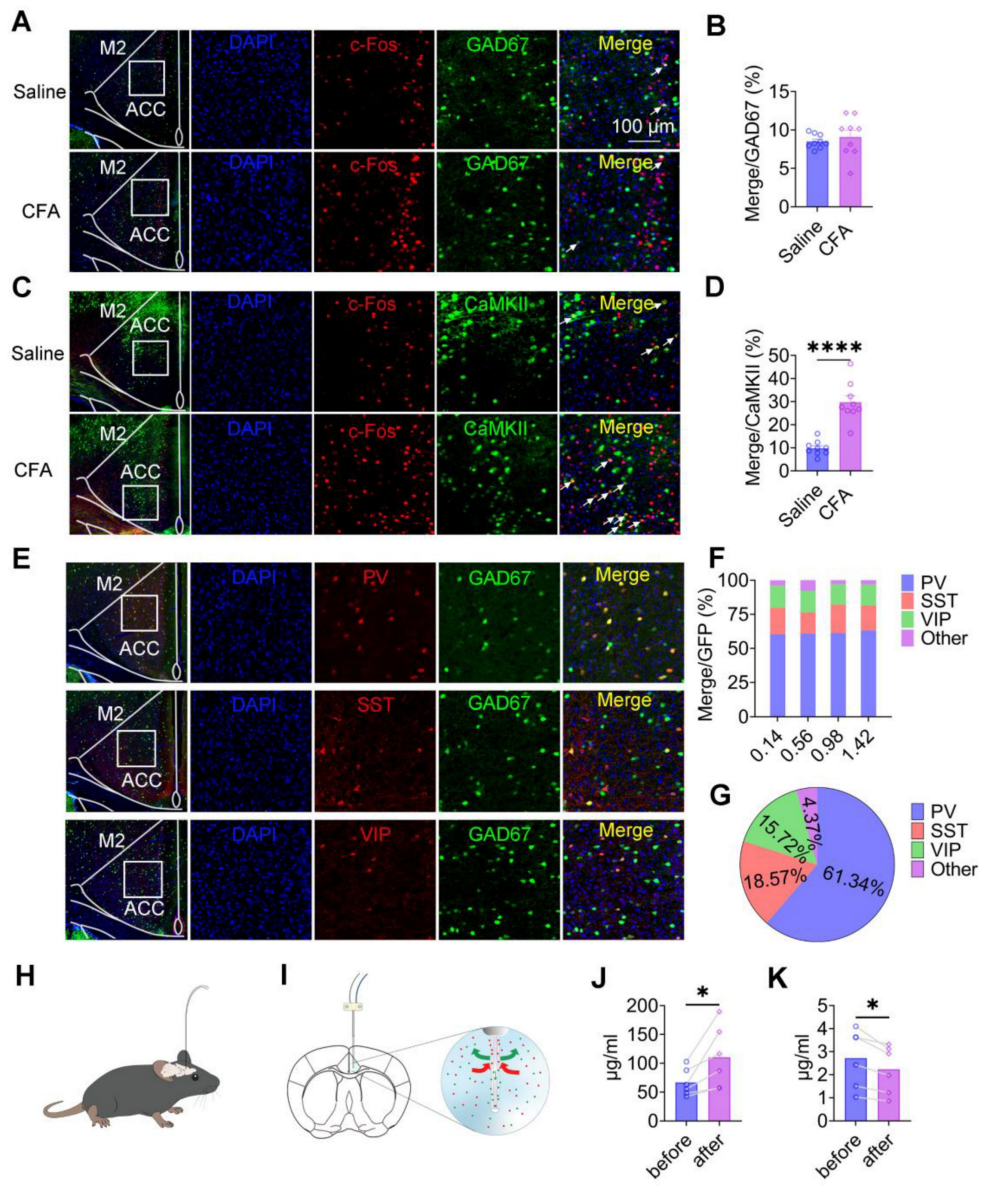
** Pain selectively activates CaMKII⁺ neurons and alters excitatory/inhibitory neurotransmission in the ACC.** (A) Representative images of GAD67 and c-Fos immunofluorescence in the ACC of saline- and CFA-treated mice (Scale bar = 100 μm). (B) Quantification of c-Fos^+^ cells among GAD67^+^ neurons in saline- and CFA-treated mice (Saline: n = 9, CFA: n = 9, *P* = 0.5275). (C) Representative images of CaMKII^+^ neurons and c-Fos immunofluorescence in the ACC of saline- and CFA-treated mice (Scale bar = 100 μm). (D) Quantification of c-Fos^+^ cells among CaMKII^+^ neurons in saline- and CFA-treated mice (Saline: n = 9, CFA: n = 9, *P* < 0.0001). (E) Representative immunofluorescence images of GAD67^+^, PV^+^ (top), SST^+^ (middle), and VIP^+^ (bottom) neurons in the ACC (Scale bar = 100 μm). (F) Distribution of PV^+^, SST^+^, VIP^+^, and other GABAergic neuron subtypes along the rostrocaudal axis of the ACC. (G) Proportional composition of PV^+^, SST^+^, VIP^+^, and other GABAergic neuron subtypes in the ACC. (H-I) Schematic illustrations of the microdialysis setup and probe placement in the ACC. (J) Extracellular glutamate concentrations in the ACC following CFA treatment. (K) Extracellular GABA concentrations in the ACC following CFA treatment. Data are presented as mean ± SEM. Scale bars, statistical tests, and *n* values are indicated in the panels.

To determine how pain affects neuronal activity within the ACC, we analyzed the expression of c-Fos as a marker of neuronal activation in both inhibitory and excitatory neuronal populations. Quantitative immunofluorescence analysis revealed that the proportion of c-Fos-positive GAD67⁺ neurons did not differ between saline-treated and CFA-treated mice (Figures 3A-B), indicating that inflammatory pain does not broadly activate inhibitory neurons in the ACC. In contrast, in wild-type mice injected with rAAV-CaMKIIα-EGFP to label CaMKII^+^ neurons, CFA injection resulted in a marked increase in c-Fos expression within CaMKII⁺ neurons 2 days after treatment (Figures 3C-D). These findings indicate that inflammatory pain preferentially activates excitatory pyramidal neurons in the ACC, without a corresponding increase in inhibitory neuron activation. To further characterize pain-related effects on inhibitory microcircuits, we examined the distribution and activation of major GABAergic interneuron subtypes, including PV⁺, SST⁺, and VIP⁺ neurons (Figure 3E). These interneuron populations were evenly distributed along the rostrocaudal extent of the ACC (anterior-posterior coordinates: 0.14-1.42 mm; Figure 3F). Quantitative analysis revealed that PV⁺, SST⁺, and VIP⁺ neurons accounted for 61.34%, 18.57%, and 15.72% of the total GABAergic population, respectively, whereas other interneuron subtypes comprised 4.37% (Figure 3G). We next assessed whether inflammatory pain selectively activated specific GABAergic interneuron subtypes. Immunofluorescence analysis of c-Fos expression revealed no significant differences in c-Fos overlap with PV⁺, SST⁺, or VIP⁺ neurons between saline- and CFA-treated mice (Figures S4A-F), indicating that inflammatory pain does not selectively recruit specific inhibitory interneuron subpopulations in the ACC. Finally, to determine whether pain alters excitatory and inhibitory neurotransmission at the population level, we performed *in vivo* microdialysis in freely moving mice (Figures 3H-I). HPLC analysis revealed a robust increase in extracellular glutamate levels (Figure 3J) accompanied by a significant reduction in extracellular GABA levels (Figure 3K) following CFA injection. Together, these findings demonstrate that inflammatory pain induces a shift toward excitatory dominance in the ACC, driven by enhanced activation of CaMKII⁺ neurons and altered excitatory/inhibitory neurotransmitter balance.

### Bidirectional modulation of ACC GABAergic neurons differentially alleviates pain and social deficits

**Figure 4:**
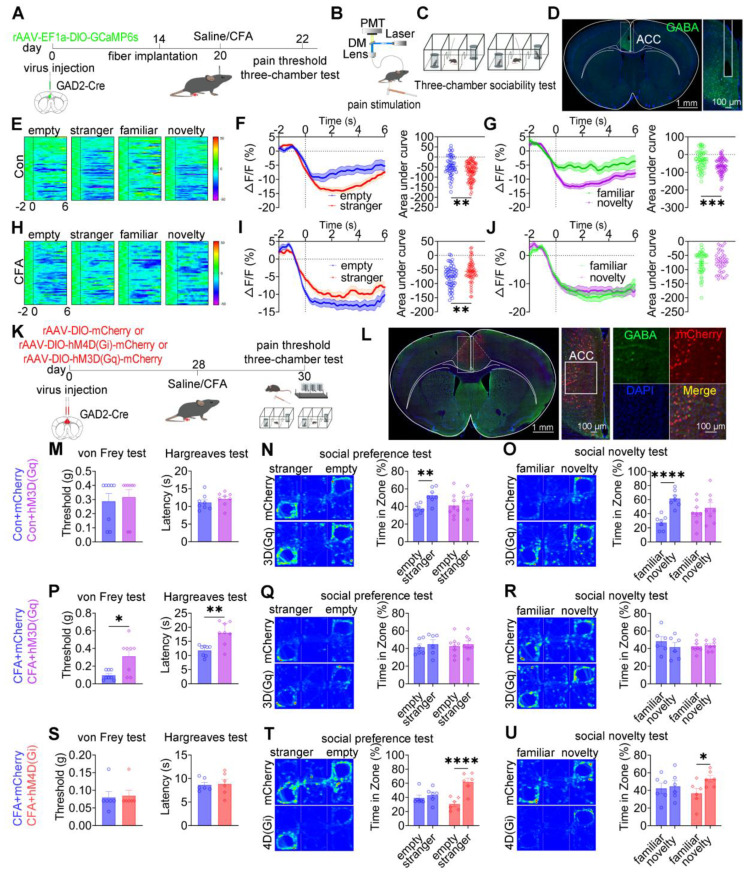
** Bidirectional modulation of ACC GABAergic neurons differentially regulates pain and social behavior.** (A) Fiber photometry experimental workflow. (B-C) Schematics of photometry during mechanical stimulation and three-chamber testing. (D) Immunofluorescence confirming GCaMP6s expression specificity in GAD2-Cre mice. Scale bar = 100 μm. (E) Heatmap of calcium activity in ACC GABAergic neurons during social interaction in sham mice. (F) Peri-event plots of calcium signals during social preference in sham mice (empty: n = 52 trials from six mice, stranger: n = 85 trials from six mice). (G) Peri-event plots of calcium signals during social novelty in sham mice (familiar: n = 53 trials from six mice, novelty: n = 72 trials from six mice). (H) Heatmap of calcium activity during social interaction in CFA. (I) Peri-event plots of calcium signals during social preference in CFA mice (empty: n = 56 trials from six mice; stranger: n = 60 trials from six mice). (J) Peri-event plots of calcium signals during social novelty in CFA mice (familiar: n = 42 trials from six mice, novelty: n = 38 trials from six mice). (K) Experimental timeline for chemogenetic manipulation. (L) Immunofluorescence confirming hM3D(Gq) or hM4D(Gi) expression in GAD2-Cre mice. Scale bar = 50 μm. (M) Mechanical and thermal pain thresholds in sham mice following chemogenetic activation. Mechanical pain (mCherry: n = 8, hM3D(Gq): n = 8) and thermal pain (mCherry: n = 8, hM3D(Gq): n = 8). (N-O) Heatmaps and quantification of social preference and social novelty in sham mice following hM3D(Gq) activation. (mCherry: n = 7, hM3D(Gq): n = 8). (P) Pain thresholds in CFA mice following hM3D(Gq) activation (mCherry: n = 8, hM3D(Gq): n = 8). (Q-R) Heatmaps and quantification of social preference and social novelty in CFA mice following hM3D(Gq) activation (mCherry: n = 6, hM3D(Gq): n = 8). (S) Pain thresholds in CFA mice following hM4D(Gi) inhibition (mCherry: n = 6, hM4D(Gi): n = 6). (T-U) Heatmaps and quantification of social preference and social novelty in CFA mice following hM4D(Gi) inhibition (mCherry: n = 6, hM4D(Gi): n = 6). Data are presented as mean ± SEM. Scale bars, *n* values, and statistical tests are indicated in the panels.

To investigate the role of ACC GABAergic neuronal activity in pain processing and social behavior, fiber photometry was performed in GAD2-Cre mice expressing GCaMP6s in ACC GABAergic neurons. A schematic overview of the experimental design is shown in Figures 4A-D. In saline-treated mice, stimulation with a low-intensity von Frey filament (0.07 g) did not evoke pain-like responses or calcium transients, whereas high-intensity stimulation (1.0 g) elicited both nociceptive responses and increased calcium activity in ACC GABAergic neurons (Figures S5A-C). In contrast, in CFA-treated mice, both low- and high-intensity mechanical stimulation induced pain responses and robust increases in calcium signaling (Figures S5D-F), indicating heightened sensitivity of ACC GABAergic neurons under inflammatory pain conditions. During social interaction, saline-treated mice exhibited a consistent decrease in ACC GABAergic neuronal activity when approaching social targets, including the empty cage, stranger, familiar, and novelty mice (Figure 4E). Both social preference (stranger vs. empty cage; Figure 4F) and social novelty (novel vs. familiar; Figure 4G) were associated with a greater reduction in calcium signals. In CFA-treated mice, although reduced GABAergic activity near social targets was preserved (Figure 4H), the dynamic modulation of calcium signals during the social preference phase (Figure 4I) and social novelty phase (Figure 4J) was disrupted. Direct comparison of calcium dynamics between saline- and CFA-treated mice revealed that CFA-treated mice exhibited significantly lower calcium signals when approaching the empty cage and familiar mice (Figures S5G-I and S5M-O). In contrast, calcium responses during approach to stranger or novel mice did not differ significantly between groups (Figures S5J-L and S5P-R). These findings indicate that inflammatory pain selectively impairs the encoding of specific social cues by ACC GABAergic neurons, thereby contributing to deficits in social preference and social novelty.

We next examined the causal role of ACC GABAergic neurons in pain and social deficits using chemogenetic and optogenetic manipulations (Figures 4K-L). In sham mice, chemogenetic activation of ACC GABAergic neurons via hM3D(Gq) or inhibition via hM4D(Gi) did not alter mechanical or thermal pain thresholds (Figures 4M and S6A). However, hM3D(Gq)-mediated activation induced deficits in both social preference and social novelty (Figures 4N-O), whereas hM4D(Gi)-mediated inhibition did not affect social behavior (Figures S6B-C). In CFA-treated mice, hM3D(Gq) activation significantly increased mechanical and thermal pain thresholds but failed to rescue social deficits (Figures 4P-R). In contrast, hM4D(Gi) inhibition restored both social preference and social novelty behaviors without affecting pain sensitivity (Figures 4S-U). Similar patterns were observed in SNI mice: hM3D(Gq) activation alleviated mechanical allodynia but did not improve social deficits (Figures S6D-F), whereas hM4D(Gi) inhibition rescued social behavior independently of nociceptive modulation (Figures S6G-I).

Optogenetic manipulation produced consistent results. In sham mice, ChR2-mediated activation of ACC GABAergic neurons induced social deficits without altering pain thresholds (Figures S7A-C), whereas eNpHR-mediated inhibition had no effect on either pain or social behavior (Figures S7D-F). In CFA-treated mice, ChR2 activation increased pain thresholds but did not improve social deficits (Figures S7G-I), while eNpHR inhibition restored social preference and novelty without affecting nociception (Figures S7J-L). SNI mice exhibited comparable outcomes, with ChR2 activation reducing mechanical allodynia but not social deficits (Figures S7M-O) and eNpHR inhibition selectively rescuing social behavior (Figures S7P-R). Collectively, these findings demonstrate that ACC GABAergic neurons exert bidirectional and dissociable control over pain and social behavior: activation preferentially modulates nociception, whereas inhibition selectively rescues pain-induced social deficits.

### ACC PV^+^ neurons bidirectionally modulate pain and social preference but not social novelty

**Figure 5:**
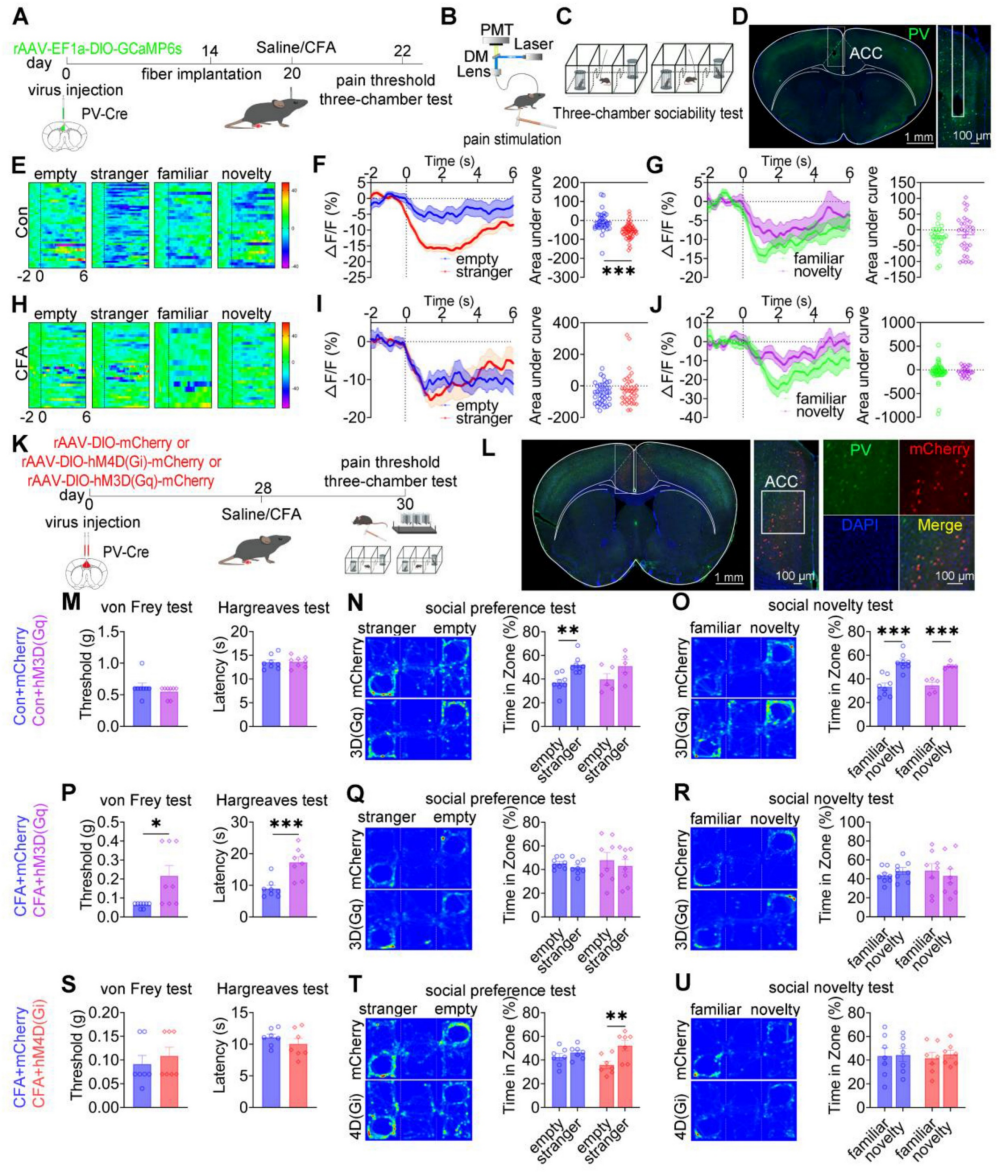
** ACC PV^+^ neurons bidirectionally regulate pain and social preference but not social novelty.** (A) Fiber photometry experimental workflow. (B-C) Schematics of photometry during mechanical stimulation and three-chamber testing. (D) Immunofluorescence confirming GCaMP6s expression specificity in PV-Cre mice. Scale bars = 100 μm. (E) Heatmap of calcium activity in ACC PV^+^ neurons during social interaction in sham mice. (F) Peri-event plots of calcium signals during social preference in sham mice (empty: n = 35 trials from six mice, stranger: n = 48 trials from six mice). (G) Peri-event plots of calcium signals during social novelty in sham mice (familiar: n = 28 trials from six mice, novelty: n = 32 trials from six mice). (H) Heatmap of calcium activity during social interaction. (I) Peri-event plots of calcium signals during social preference in CFA mice (empty: n = 38 trials from six mice; stranger: n = 38 trials from six mice). (J) Peri-event plots of calcium signals during social novelty in CFA mice (familiar: n = 49 trials from six mice, novelty: n = 24 trials from six mice). (K) Experimental timeline for chemogenetic manipulation. (L) Immunofluorescence confirming hM4D(Gi) or hM3D(Gq) expression in PV-Cre mice. Scale bars = 50 μm. (M) Mechanical and thermal pain thresholds in sham mice following hM3D(Gq) activation (mCherry: n = 8, hM3D(Gq): n = 8). (N-O) Heatmaps and quantification of social preference and social novelty in sham mice following hM3D(Gq) activation (mCherry: n = 8, hM3D(Gq): n = 5). (P) Pain thresholds in CFA mice following hM3D(Gq) activation (mCherry: n = 8, hM3D(Gq): n = 8). (Q-R) Heatmaps and quantification of social preference and social novelty in CFA mice following hM3D(Gq) activation (mCherry: n = 8, hM3D(Gq): n = 8). (S) Pain thresholds in CFA mice following hM4D(Gi) inhibition (mCherry: n = 7, hM4D(Gi): n = 7). (T-U) Heatmaps and quantification of social preference and social novelty in CFA mice following hM4D(Gi) inhibition (mCherry: n = 7, hM4D(Gi): n = 7). Data are presented as mean ± SEM. Scale bars, *n* values, and statistical tests are indicated in the panels.

To dissect the role of ACC PV^+^ neurons in pain processing and social behavior, fiber photometry was performed in PV-Cre mice expressing GCaMP6s selectively in these neurons (Figures 5A-D). In saline-treated mice, low-intensity mechanical stimulation (0.07 g von Frey filament) failed to elicit nociceptive responses or detectable calcium transients, whereas high-intensity stimulation (1.0 g) induced robust pain responses accompanied by increased PV⁺ neuronal activity (Figures S8A-C). In CFA-treated mice, both low- and high-intensity mechanical stimuli evoked hypersensitivity and pronounced activation of PV⁺ neurons (Figures S8D-F).

During social interaction, saline-treated mice exhibited reduced PV^+^ neuronal activity when approaching social targets, including the empty cage, stranger, familiar, and novel mice (Figure 5E). Social preference behavior (stranger vs empty cage) was associated with a significant reduction in calcium signals (Figure 5F), whereas social novelty behavior (novel vs familiar) did not induce significant modulation of PV^+^ neuronal activity (Figure 5G). In CFA-treated mice, although reduced PV^+^ activity near social targets was preserved, dynamic signal modulation during the social preference phase was disrupted, while responses during social novelty remained unchanged (Figures 5H-J). Direct comparison of calcium dynamics between saline- and CFA-treated mice revealed that CFA-treated mice exhibited significantly lower PV⁺ neuronal activity when approaching the empty cage (Figures S8G-I). In contrast, calcium signals did not differ between groups during approaches to stranger, familiar, or novel mice (Figures S8J-R). These findings indicate that ACC PV^+^ neurons preferentially encode social preference, and that this encoding is selectively impaired under inflammatory pain conditions.

We next examined the causal contribution of ACC PV^+^ neurons to pain-induced social deficits using chemogenetic manipulation (Figures 5K-L). In sham mice, chemogenetic activation of PV^+^ neurons via hM3D(Gq) did not alter mechanical or thermal pain thresholds (Figure 5M) but induced deficits in social preference without affecting social novelty (Figures 5N-O). In contrast, hM4D(Gi)-mediated inhibition spared both nociceptive sensitivity and social behavior (Figures S9A-C). In CFA-treated mice, hM3D(Gq) activation significantly alleviated mechanical and thermal hyperalgesia (Figure 5P) but failed to rescue deficits in either social preference or social novelty (Figures 5Q-R). Conversely, hM4D(Gi) inhibition selectively restored social preference while leaving social novelty deficits and pain sensitivity unchanged (Figures 5S-U). Similar patterns were observed in SNI mice, in which hM3D(Gq) activation alleviated mechanical allodynia without improving social deficits (Figures S9D-F), whereas hM4D(Gi) inhibition selectively rescued social preference while sparing pain thresholds and social novelty deficits (Figures S9G-I). Collectively, these results demonstrate that ACC PV**^+^** neurons bidirectionally regulate pain and social preference, but do not mediate social novelty processing.

### ACC SST^+^ neurons modulate social novelty deficits independent of pain perception

**Figure 6:**
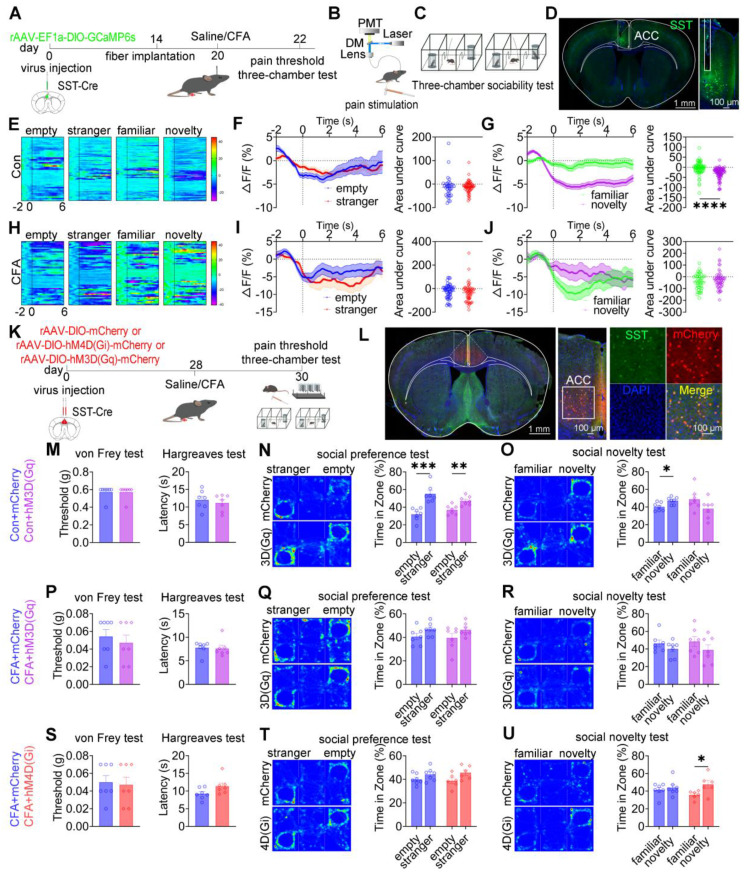
** ACC SST^+^ neurons selectively regulate social novelty independent of pain.** (A) Fiber photometry experimental workflow. (B-C) Schematics of photometry during mechanical stimulation and three-chamber testing. (D) Immunofluorescence confirming GCaMP6s expression specificity in SST-Cre mice. Scale bars = 100 μm. (E) Heatmap of calcium activity in ACC SST^+^ neurons during social interaction in sham mice during. (F) Peri-event plots of calcium signals during social preference in sham mice (empty: n = 35 trials from six mice, stranger: n = 61 trials from six mice). (G) Peri-event plots of calcium signals during social novelty in sham mice (familiar: n = 70 trials from six mice, novelty: n = 86 trials from six mice). (H) Heatmap of calcium activity during social interaction in CFA mice. (I) Peri-event plots of calcium signals during social preference in CFA mice (empty: n = 52 trials from six mice, stranger: n = 56 trials from six mice). (J) Peri-event plots of calcium signals during social novelty in CFA mice (familiar: n = 46 trials from six mice, novelty: n = 51 trials from six mice). (K) Experimental timeline for chemogenetic manipulation. (L) Immunofluorescence confirming hM3D(Gq) or hM4D(Gi) expression in SST-Cre mice. Scale bars = 50 μm. (M) Mechanical and thermal pain thresholds in sham mice following hM3D(Gq) activation (mCherry: n = 7, hM3D(Gq): n = 7). (N-O) Heatmaps and quantification of social preference and social novelty in sham mice following hM3D(Gq) activation (mCherry: n = 7, hM3D(Gq): n = 7). (P) Pain thresholds in CFA mice following hM3D(Gq) activation (mCherry: n = 7, hM3D(Gq): n = 7). (Q-R) Heatmaps and quantification of social preference and social novelty in CFA mice following hM3D(Gq) activation (mCherry: n = 7, hM3D(Gq): n = 7). (S) Pain thresholds in CFA mice following hM4D(Gi) inhibition (mCherry: n = 7, hM4D(Gi): n = 7. (T-U) Heatmaps and quantification of social preference and social novelty in CFA mice following hM4D(Gi) inhibition (mCherry: n = 7, hM4D(Gi): n = 7). Data are presented as mean ± SEM. Scale bars, *n* values, and statistical tests are indicated in the panels.

To examine the role of ACC SST^+^ neurons in nociception and social behavior, fiber photometry was performed in SST-Cre mice expressing GCaMP6s during mechanical pain testing and three-chamber social interaction assays (Figures 6A-D). In saline-treated mice, subthreshold mechanical stimulation (0.07 g) von Frey filament failed to evoke pain-like behaviors or detectable calcium transients, whereas suprathreshold stimulation (1.0 g) induced nociceptive responses accompanied by increased SST⁺ neuronal activity (Figures S10A-C). In CFA-treated mice, both low- and high-intensity mechanical stimuli elicited robust pain responses and elevated SST⁺ neuronal activity (Figures S10D-F). During social interaction, saline-treated mice exhibited decreased SST⁺ neuronal activity when approaching social targets, including the empty cage, stranger, familiar, and novel mice (Figure 6E). During the social preference phase, the magnitude of calcium signal reduction did not differ between approaches to the stranger and the empty cage (Figure 6F). In contrast, during the social novelty phase, the reduction in SST⁺ neuronal activity was significantly greater when approaching novel mice compared with familiar mice (Figure 6G). In CFA-treated mice, although baseline reductions in SST⁺ neuronal activity near social targets were preserved, modulation of calcium signals during the social novelty phase was disrupted, while responses during social preference remained unchanged (Figures 6H-J). Direct comparison of calcium dynamics between saline- and CFA-treated mice revealed that CFA-treated mice exhibited significantly lower SST^+^ neuronal activity when approaching stranger and familiar mice (Figures S10J-O). In contrast, calcium signals did not differ between groups when approaching the empty cage or novel mice (Figures S10G-I and S10P-R). These findings indicate that ACC SST^+^ neurons preferentially encode social novelty information and that inflammatory pain selectively disrupts this encoding process.

To establish a causal relationship, chemogenetic manipulation of ACC SST^+^ neurons was performed (Figures 6K-L). In sham mice, activation of SST^+^ neurons via hM3D(Gq) did not alter mechanical or thermal pain thresholds or social preference but induced pronounced social novelty deficits (Figures 6M-O). Conversely, hM4D(Gi)-mediated inhibition did not affect nociceptive sensitivity or social behavior (Figures S11A-C). In CFA-treated mice, neither hM3D(Gq) activation nor hM4D(Gi) inhibition altered mechanical or thermal hyperalgesia (Figures 6P and 6S). Notably, hM4D(Gi)-mediated inhibition selectively rescued social novelty deficits while leaving social preference deficits unchanged (Figures 6Q-R and 6T-U). Consistent with these findings, hM4D(Gi)-treated SNI mice exhibited restored social novelty behavior without improvement in social preference (Figures S11G-I). Conversely, hM3D(Gq) activation in SNI mice did not exacerbate either pain sensitivity or social deficits (Figures S11D-F). Together, these results demonstrate that ACC SST⁺ neurons selectively regulate social novelty processing independent of pain perception, and that inhibition of this neuronal population is sufficient to rescue pain-induced social novelty deficits.

### ACC VIP^+^ neurons are not engaged in pain processing or social behavior

**Figure 7:**
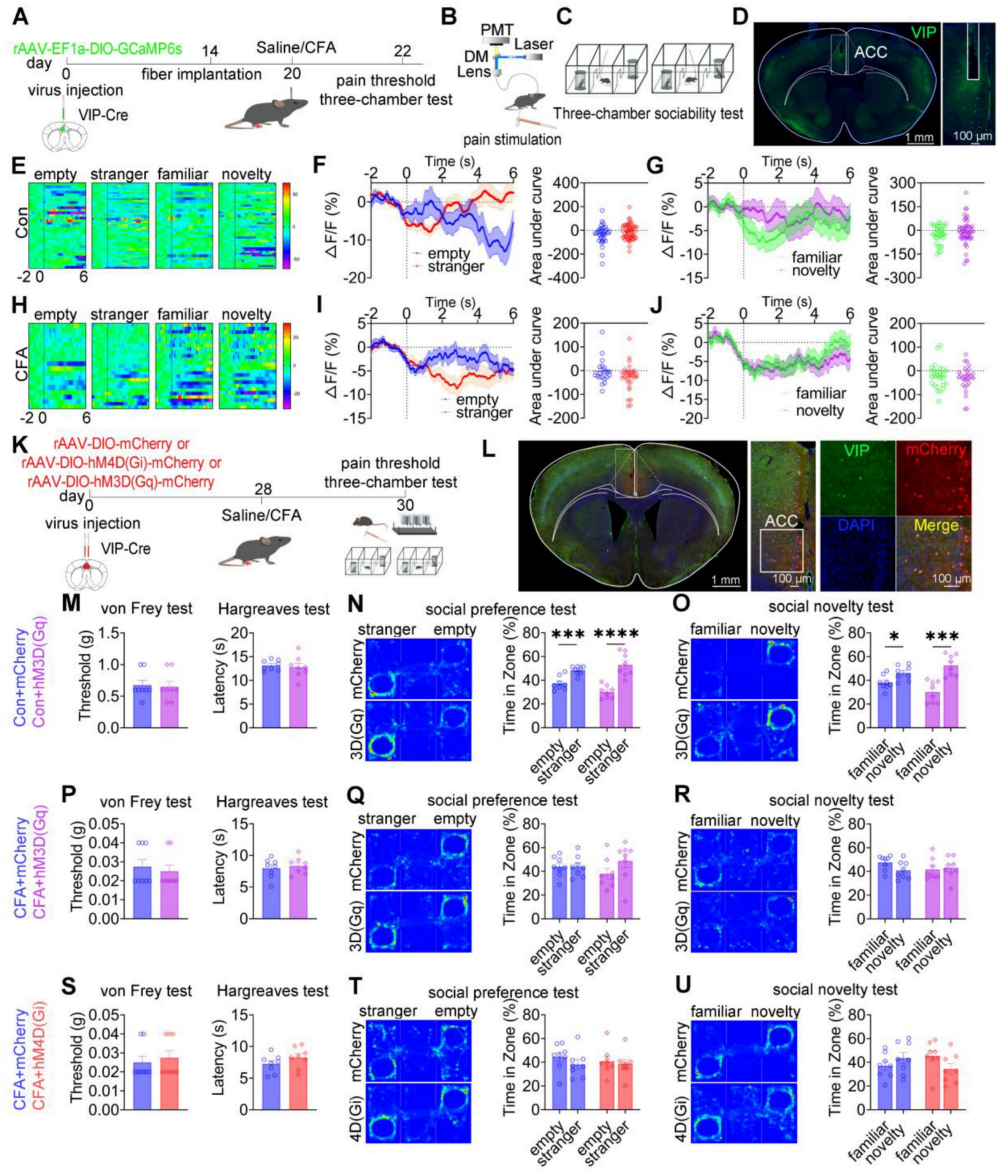
** ACC VIP^+^ neurons do not regulate pain or social behavior.** (A) Fiber photometry experimental workflow. (B-C) Schematics of photometry during mechanical stimulation and three-chamber testing. (D) Immunofluorescence confirming GCaMP6s expression specificity in VIP-Cre mice. Scale bars = 100 μm. (E) Heatmap of calcium activity in ACC VIP⁺ neurons during social interaction in sham mice. (F) Peri-event plots of calcium signals during social preference in sham mice (empty: n = 33 trials from four mice, empty: n = 57 trials from four mice). (G) Peri-event plots of calcium signals during social novelty in sham mice (familiar: n = 30 trials from four mice, novelty: n = 51 trials from four mice). (H) Heatmap of calcium activity during social interaction in CFA mice. (I) Peri-event plots of calcium signals during social preference in sham mice (empty: n = 20 trials from four mice, stranger: n = 31 trials from four mice). (J) Peri-event plots of calcium signals during social novelty in CFA mice (familiar: n = 26 trials from four mice, novelty: n = 28 trials from four mice). (K) Experimental timeline for chemogenetic manipulation. (L) Immunofluorescence confirming hM4D(Gi) or hM3D(Gq) expression in VIP-Cre mice. Scale bars = 50 μm. (M) Mechanical and thermal pain thresholds in sham mice following hM3D(Gq) activation (mCherry: n = 8, hM3D(Gq): n = 8). (N-O) Heatmaps and quantification of social preference and social novelty in sham mice following hM3D(Gq) activation (mCherry: n = 8, hM3D(Gq): n = 8). (P) Pain thresholds in CFA mice following hM3D(Gq) activation (mCherry: n = 8, hM3D(Gq): n = 8). (Q-R) Heatmaps and quantification of social preference and social novelty in CFA mice following hM3D(Gq) activation (mCherry: n = 8, hM3D(Gq): n = 8). (S) Pain thresholds in CFA mice following hM4D(Gi) inhibition (mCherry: n = 8, hM4D(Gi): n = 8). (T-U) Heatmaps and quantification of social preference and social novelty in CFA mice following hM4D(Gi) inhibition (mCherry: n = 8, hM4D(Gi): n = 8). Data are presented as mean ± SEM. Scale bars, *n* values, and statistical tests are indicated in the panels.

To examine whether ACC VIP⁺ neurons contribute to pain processing or social behavior, calcium activity was recorded in VIP-Cre mice expressing GCaMP6s during mechanical nociceptive stimulation and three-chamber social interaction tasks (Figures 7A-D). In saline-treated mice, mechanical stimulation with either a subthreshold (0.07 g) or suprathreshold (1.0 g) von Frey filament failed to induce detectable changes in VIP^+^ neuronal calcium activity (Figures S12A-C). Although CFA-treated mice exhibited robust hypersensitivity to both stimulus intensities, VIP^+^ neuronal activity remained unaltered during nociceptive stimulation (Figures S12D-F). During social interaction, saline-treated mice showed stable VIP⁺ neuronal activity when approaching social targets, including the empty cage, stranger, familiar, and novel mice (Figures 7E-G). Similarly, despite exhibiting deficits in social preference and social novelty, CFA-treated mice showed no significant modulation of VIP⁺ neuronal calcium signals during either the social preference phase (stranger vs empty cage) or the social novelty phase (novel vs familiar; Figures 7H-J). Direct comparison of calcium dynamics between saline- and CFA-treated mice revealed no significant differences in VIP⁺ neuronal activity when approaching any social target (Figures S12G-R). These findings indicate that ACC VIP^+^ neurons do not encode nociceptive or social behavioral information under either physiological or inflammatory pain conditions.

To directly test causality, chemogenetic activation or inhibition of ACC VIP⁺ neurons was performed (Figures 7K-L). In saline-treated mice, neither chemogenetic activation [hM3D(Gq)] nor inhibition [hM4D(Gi)] altered mechanical or thermal pain thresholds, nor did it affect social preference or social novelty behaviors compared with mCherry controls (Figures 7M-O and S13A-C). Similarly, in CFA-treated mice, neither activation nor inhibition of VIP⁺ neurons modified pain hypersensitivity or rescued social deficits (Figures 7P-U). Consistent results were observed in the SNI model, in which chemogenetic manipulation of VIP^+^ neurons failed to affect nociceptive sensitivity or social behavior (Figures S13D-I).

### ACC CaMKII^+^ neurons bidirectionally modulate pain and social preference but not social novelty

Having demonstrated that ACC GABAergic neurons play critical roles in pain processing and social behavior regulation, and given evidence that maintenance of excitatory/inhibitory (E/I) balance within the ACC is essential for normal social behavior, we hypothesized that GABAergic neurons regulate pain and social behavior by dynamically modulating local CaMKII⁺ excitatory neuronal activity. Because excitatory neurons constitute the majority of ACC neurons and E/I imbalance has been closely linked to social deficits in neurodevelopmental disorders, elucidating GABAergic-excitatory interactions is essential for understanding ACC circuit mechanisms underlying pain-induced social deficits. To test this hypothesis, *in vivo* fiber photometry was used to monitor real-time modulation of CaMKII⁺ neuronal activity by GABAergic neurons in the ACC (Figures S14A-C). Optogenetic suppression of GABAergic neurons using 594 nm light at 5 mW resulted in a significant increase (~7%) in CaMKII⁺ neuronal calcium signals. Increasing light power to 15 mW further potentiated this effect, producing an approximately 12% increase in calcium activity (Figures S14D-F). This dose-dependent disinhibition indicates that ACC GABAergic neurons finely tune excitatory neuronal activity through graded inhibitory control, thereby maintaining E/I balance. These findings suggest that CaMKII⁺ neurons, as a principal excitatory component of this balance, may play distinct roles in pain perception and social behavior.

**Figure 8:**
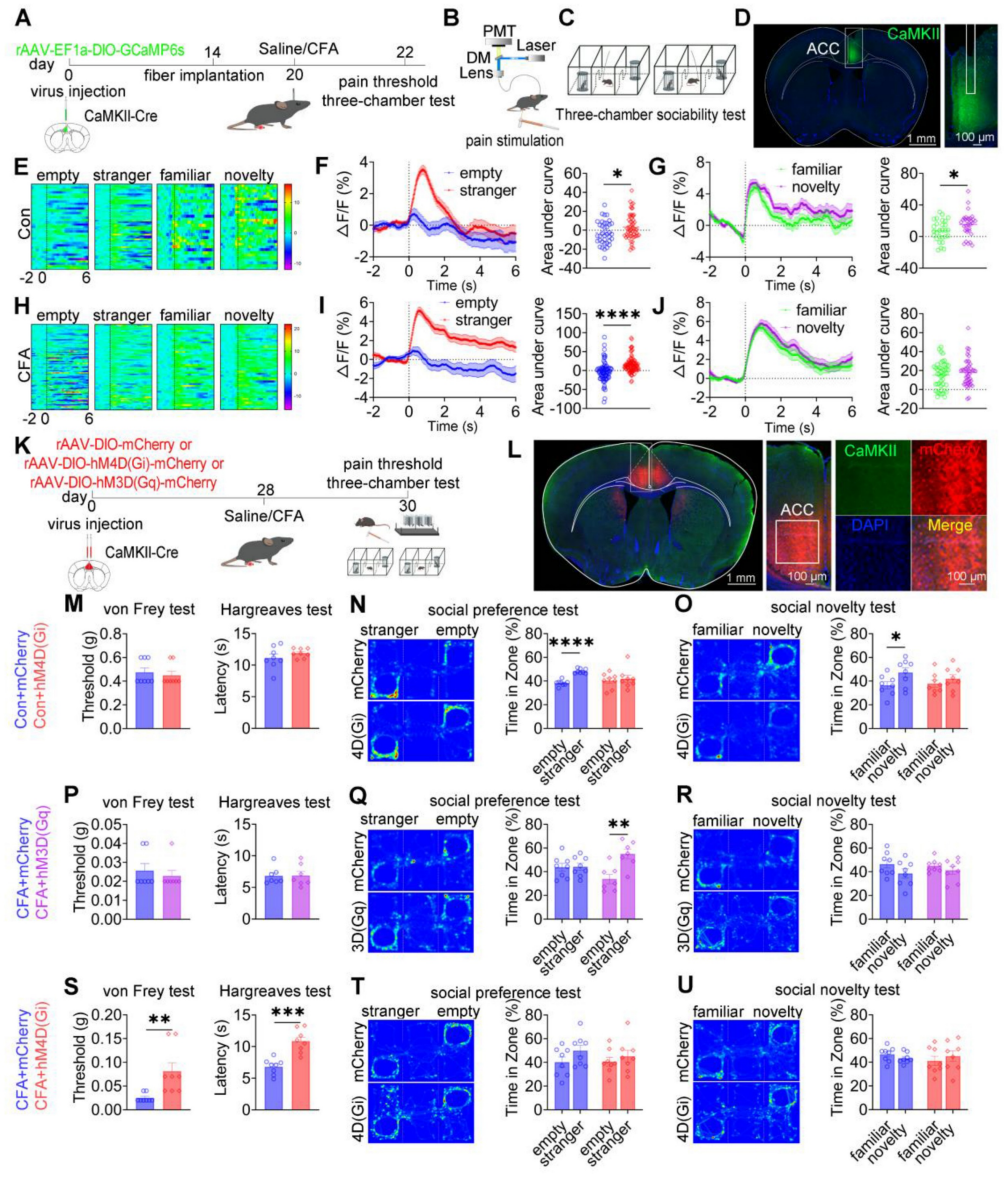
** ACC CaMKII^+^ neurons bidirectionally regulate pain and social preference but not social novelty.** (A) Fiber photometry experimental workflow. (B-C) Schematics of photometry during mechanical stimulation and three-chamber testing. (D) Immunofluorescence confirming GCaMP6s expression specificity in CaMKII-Cre mice. Scale bars = 100 μm. (E) Heatmap of calcium activity in ACC CaMKII⁺ neurons during social interaction in sham mice. (F) Peri-event plots of calcium signals during social preference in sham mice (empty: n = 37 trials from five mice, stranger: n = 55 trials from five mice). (G) Peri-event plots of calcium signals during social novelty in sham mice (familiar: n = 28 trials from five mice, novelty: n = 32 trials from five mice). (H) Heatmap of calcium activity during social interaction in sham mice. (I) Peri-event plots of calcium signals during social preference in sham mice (empty: n = 78 trials from five mice, stranger: n = 98 trials from five mice). (J) Peri-event plots of calcium signals during social novelty phase in CFA mice (familiar: n = 45 trials from five mice, novelty: n = 45 trials from five mice). (K) Experimental timeline for chemogenetic manipulation. (L) Immunofluorescence confirming hM3D(Gq) or hM4D(Gi) expression in CaMKII-Cre mice. Scale bars = 50 μm. (M) Mechanical and thermal pain thresholds in sham mice following hM4D(Gi) inhibition (mCherry: n = 8, hM4D(Gi): n = 8). (N-O) Heatmaps and quantification of social preference and social novelty following hM4D(Gi) inhibition (mCherry: n = 8, hM4D(Gi): n = 8). (P) Pain thresholds in CFA mice following hM3D(Gq) activation (mCherry: n = 7, hM3D (Gq): n = 7). (Q-R) Heatmaps and quantification of social preference and social novelty following hM3D(Gq) activation (mCherry: n = 8, hM3D(Gq): n = 7). (S) Pain thresholds in CFA mice following hM4D(Gi) inhibition (mCherry: n = 8, hM4D(Gi): n = 8). (T-U) Heatmaps and quantification of social preference and social novelty following hM4D(Gi) inhibition (mCherry: n = 8, hM4D(Gi): n = 8). Data are presented as mean ± SEM. Scale bars, *n* values, and statistical tests are indicated in the panels.

To directly examine the role of ACC CaMKII⁺ neurons, fiber photometry was performed in CaMKII-Cre mice expressing GCaMP6s in the ACC (Figures 8A-D). In saline-treated mice, subthreshold mechanical stimulation (0.07 g) elicited neither nociceptive responses nor changes in calcium signals, whereas suprathreshold stimulation (1.0 g) induced robust pain responses accompanied by increased CaMKII^+^ neuronal activity (Figures S15A-C). In CFA mice, both stimulus intensities produced hypersensitivity and pronounced activation of CaMKII^+^ neurons (Figures S15D-F). During social interaction, saline-treated mice exhibited increased CaMKII⁺ neuronal activity when approaching social targets, including the empty cage, stranger, familiar, and novel mice (Figure 8E). During the social preference phase, calcium signals were significantly higher during approaches to the stranger mouse (Figure 8F). Similarly, during the social novelty phase, CaMKII⁺ neuronal activity was significantly elevated when approaching the novel mouse compared with the familiar mouse (Figure 8G). In CFA-treated mice, elevated CaMKII⁺ neuronal activity near social targets was preserved (Figure 8H); however, signal modulation during social preference was absent (Figure 8I), and social novelty-associated modulation was disrupted (Figure 8J). Comparative analysis revealed that CFA-treated mice exhibited significantly higher CaMKII⁺ neuronal activity than saline controls when approaching stranger and familiar mice (Figures S15J-O). In contrast, calcium signals did not differ between groups when approaching the empty cage or novel mice (Figures S15G-I and S15P-R). These results indicate that CaMKII⁺ neurons encode social behavioral information and that inflammatory pain selectively disrupts this encoding.

To assess causality, rAAV-EF1a-DIO-mCherry, rAAV-EF1a-DIO-hM3D(Gq)-mCherry, or rAAV-EF1a-DIO-hM4D(Gi)-mCherry was injected into the ACC of CaMKII-Cre mice (Figures 8K-L). In sham mice, chemogenetic activation of CaMKII⁺ neurons did not alter baseline mechanical or thermal sensitivity (Figure S16A). While control mice displayed normal social preference and novelty, hM3D(Gq) activation preserved social preference but induced social novelty deficits (Figures S16B-C). Conversely, hM4D(Gi)-mediated inhibition did not affect baseline pain sensitivity (Figure 8M) but resulted in deficits in both social preference and social novelty (Figures 8N-O).

In CFA-treated mice, hM3D(Gq)-mediated activation failed to modify mechanical allodynia or thermal hyperalgesia (Figure 8P) but selectively rescued social preference deficits without improving social novelty deficits (Figures 8Q-R). In contrast, hM4D(Gi)-mediated inhibition significantly alleviated mechanical and thermal hypersensitivity (Figure 8S) but did not rescue deficits in social preference or novelty (Figures 8T-U). These dissociative effects were replicated in SNI mice: hM3D(Gq) activation did not alter mechanical allodynia (Figure S16D) but restored social preference without affecting social novelty (Figures S16E-F), whereas hM4D(Gi) inhibition alleviated mechanical hypersensitivity (Figure S16G) without improving social behavior (Figures S16H-I). Collectively, these findings demonstrate a clear functional dissociation within ACC CaMKII^+^ neurons, whereby distinct activity states differentially regulate pain perception and social behavior, with bidirectional control of pain and social preference but not social novelty.

### Pan-neuronal modulation of the ACC alleviates social preference deficits independently of nociceptive processing

**Figure 9:**
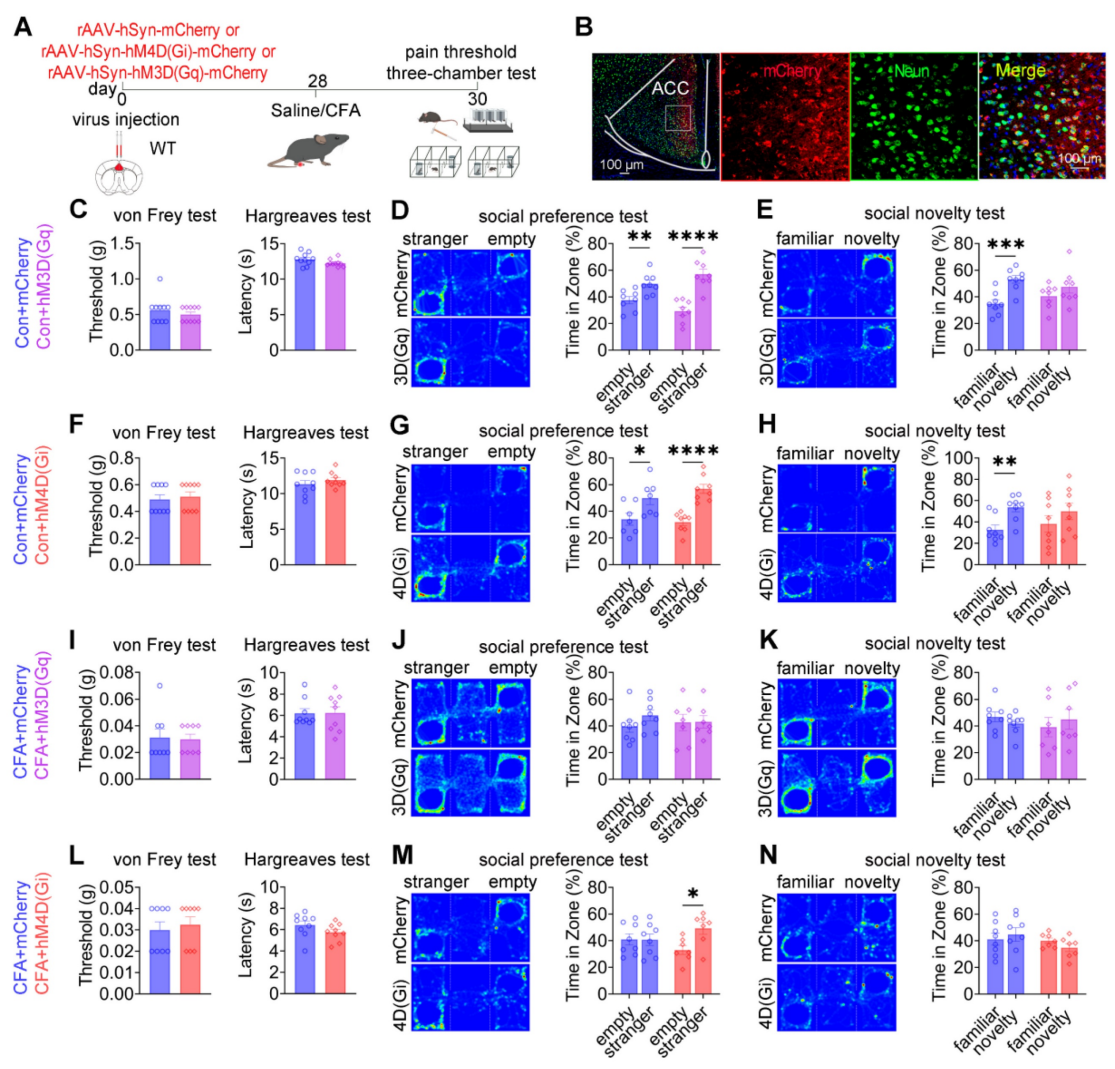
** Pan-neuronal chemogenetic modulation of the ACC selectively regulates social behavior.** (A) Experimental timeline. (B) Immunofluorescence confirming hM3D(Gq) or hM4D(Gi) expression in WT mice. Scale bars = 50 μm. (C) Mechanical and thermal pain thresholds following hM3D(Gq) activation in sham mice (mCherry: n = 10, hM3D(Gq): n = 10). (D) Heatmaps and quantification of social preference following hM3D(Gq) activation in sham mice (mCherry: n = 8, hM3D(Gq): n = 8). (E) Heatmaps and quantification of social novelty following hM3D(Gq) activation in sham mice (mCherry: n = 8, hM3D(Gq): n = 8). (F) Mechanical and thermal pain thresholds following hM4D(Gi) inhibition in sham mice (mCherry: n = 9, hM4D(Gi): n = 9). (G) Heatmaps and quantification of social preference following hM4D(Gi) inhibition in sham mice (mCherry: n = 7, hM4D(Gi): n = 8). (H) Heatmaps and quantification of social novelty following hM4D(Gi) inhibition in sham mice (mCherry: n = 8, hM4D(Gi): n = 8). (I) Mechanical and thermal pain thresholds following hM3D(Gq) activation in CFA mice (mCherry: n = 8, hM3D(Gq): n = 9). (J) Heatmaps and quantification of social preference following hM3D(Gq) activation in CFA mice (mCherry: n = 8, hM3D(Gq): n = 7). (K) Heatmaps and quantification of social novelty following hM3D(Gq) activation in CFA mice (mCherry: n = 8, hM3D(Gq): n = 7). (L) Mechanical and thermal pain thresholds following hM4D(Gi) inhibition in CFA mice (mCherry: n = 8, hM4D(Gi): n = 9). (M) Heatmaps and quantification of social preference following hM4D(Gi) inhibition in CFA mice (mCherry: n = 8, hM4D(Gi): n = 7). (N) Heatmaps and quantification of social novelty following hM4D(Gi) inhibition in CFA mice (mCherry: n = 8, hM4D(Gi): n = 7). Data are presented as mean ± SEM. Scale bars, *n* values, and statistical tests are indicated in the panels.

To assess the collective contribution of ACC neurons to pain processing and social behavior, chemogenetic manipulation was performed in wild-type mice using pan-neuronal constructs driven by the hSyn promoter: rAAV-hSyn-mCherry, rAAV-hSyn-hM3D(Gq)-mCherry, or rAAV-hSyn-hM4D(Gi)-mCherry (Figures 9A-B). In sham mice, neither chemogenetic activation nor inhibition of ACC neurons altered baseline mechanical withdrawal thresholds or thermal latency compared with mCherry controls (Figures 9C and 9F). Social behavior analysis revealed a dissociative pattern. While mCherry-expressing mice exhibited intact social preference and social novelty, both hM3D(Gq)-mediated activation and hM4D(Gi)-mediated inhibition selectively induced deficits in social novelty without affecting social preference (Figures 9D-E and 9G-H). These findings indicate that global modulation of ACC neuronal activity is sufficient to disrupt social novelty processing under baseline conditions, while sparing social preference. Under CFA-induced inflammatory pain, chemogenetic activation of ACC neurons failed to modify established mechanical allodynia, thermal hyperalgesia (Figure 9I), or the associated social deficits observed in control mice (Figures 9J-K). In contrast, chemogenetic inhibition via hM4D(Gi) produced a behaviorally selective effect: social preference deficits were fully rescued (Figure 9M), whereas mechanical allodynia, thermal hypersensitivity (Figure 9L), and social novelty deficits remained unchanged (Figure 9N). This dissociative phenotype was reproduced in the SNI model of neuropathic pain. Chemogenetic activation of ACC neurons did not alter mechanical allodynia (Figure S14A) or improve social deficits (Figures S14B-C). Conversely, ACC inhibition selectively restored social preference (Figure S14E) without affecting nociceptive sensitivity (Figure S14D) or social novelty deficits (Figure S14F). Together, these findings demonstrate that pan-neuronal inhibition of the ACC is sufficient to alleviate pain-associated social preference deficits without modifying nociceptive processing, reinforcing the dissociation between ACC-mediated regulation of social behavior and pain perception.

## Discussion

Previous studies examining pain-induced social deficits have yielded inconsistent results 14. In the present study, we systematically re-evaluated the relationship between pain and social behavior in mice and confirmed that both inflammatory and neuropathic pain robustly impair social function. Importantly, by dissecting the contributions of distinct neuronal populations within the ACC, we provide a mechanistic insight into how pain disrupts social behavior at the circuit level.

Our results demonstrate that both neuropathic and inflammatory pain reduce social preference and social novelty in mice. Notably, relatively mild inflammatory pain induced by low-dose CFA (5 µL) selectively impaired social novelty without affecting social preference, whereas higher CFA doses (10-20 µL) disrupted both behaviors. This graded effect suggests a hierarchical sensitivity of social behavior to pain intensity. Social novelty requires intact social memory and discrimination between conspecifics, processes that depend on more complex neural computations, whereas social preference reflects a more fundamental motivational drive to approach a social stimuli. Mild pain may therefore act as a cognitive stressor sufficient to disrupt fragile memory dependent processes underlying novelty recognition, while leaving basic social motivation intact. In contrast, severe pain likely induces broader motivational and affective disturbances that abolish even robust social preference.

Pain is defined as an unpleasant sensory and emotional experience associated with actual or potential tissue damage, and is frequently accompanied by negative affective states. Animal models of pain exhibit both loss of natural behaviors and emergence of abnormal behaviors 15. Social deficits in pain have often been attributed to secondary emotional disturbances such as anxiety, depression, or memory impairment. However, in the present study, social deficits were evident at early stages of pain (day 2 after CFA injection and day 5 after SNI), when anxiety-like or depression-like behaviors are not typically observed. Consistent with prior reports, mice tested shortly after CFA injection or within 2 weeks after SNI exhibit reduced pain thresholds without anxiety-like phenotypes 16, 17, while depression-like behaviors usually emerge only after prolonged neuropathic pain 18. These observations support the notion that early-stage pain can directly impair social behavior independently of emotional comorbidities. Accordingly, we focused our behavioral analyses on this early phase to minimize confounding effects of anxiety or depression.

We further observed that social deficits were reversible when pain was alleviated. As CFA-induced inflammatory pain naturally resolves, normalization of nociceptive thresholds was accompanied by recovery of social behavior. Similarly, pharmacological analgesia restored social preference without directly targeting social circuits. These findings are consistent with previous work showing that pain relief reverses social deficits in neuropathic pain models 19, and that negative affective states persist only as long as pain is present in subchronic pain paradigms 20. Notably, passive social interaction partially rescued social behavior without altering nociception, suggesting that social deficits can also be modulated independently of pain intensity. Together, these findings point to a bidirectional but asymmetric relationship between pain and social behavior.

The ACC is a key hub integrating nociceptive, emotional, and social information. Human neuroimaging studies show that diverse somatic and visceral inputs activate the ACC 21, and electrophysiological studies demonstrate enhanced ACC neuronal activity during inflammatory pain 18. Furthermore, social rejection and negative emotional states increase ACC activation 22. In our study, inflammatory pain increased excitatory neuronal activity in the ACC and disrupted inhibitory control, leading to altered excitation-inhibition (E/I) balance. Microdialysis revealed elevated extracellular glutamate and reduced GABA levels, indicating synaptic dysregulation. Although activation of ACC GABAergic neurons alleviated pain, consistent with a previous report 23, this manipulation failed to rescue pain-induced social deficits. Infusion of a GABA receptor agonist into the ACC has been shown to enhance social behavior in polyI:C-treated mice, supporting the role for ACC GABAergic signaling in social behavior regulation 24. In our study, however, post-onset activation of ACC GABAergic neurons did not improve pain-induced social deficits, whereas their inhibition selectively restored social behavior without affecting nociception. These findings reveal a functional dissociation, indicating that global manipulation of inhibitory tone of the ACC cannot simultaneously normalize nociception and social behavior.

GABAergic interneurons comprise multiple subtypes with distinct functions, among which PV^+^ and SST^+^ neurons are the most abundant 8. Our analysis confirmed that PV^+^ neurons constitute approximately 61% of ACC GABAergic neurons, followed by SST^+^ (≈19%), and VIP^+^ neurons (≈16%). Prior studies have shown reduced numbers or altered function of PV^+^ neurons in inflammatory and neuropathic pain models 25,26. Consistent with this, we found that activation of ACC PV^+^ neurons produced robust analgesia, whereas modulation of SST^+^ neurons did not affect pain sensitivity. These findings align with previous studies showing that PV^+^ neurons, which target pyramidal cell somata and proximal dendrites, are critical for inhibitory control of excitatory output and pain modulation in the ACC 11, 27. In contrast, SST^+^ neurons primarily regulate dendritic integration rather that direct output 9.

**Figure 10:**
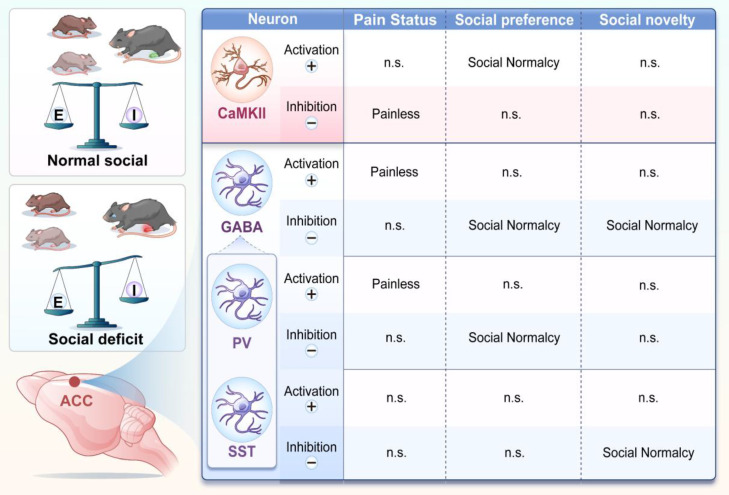
** Chronic pain remodels ACC neural circuits and disrupts E/I balance.** Schematic summary illustrating subtype-specific roles of ACC neurons in pain and social behavior. In pain states, CaMKII⁺ neuron activation rescues social preference deficits, whereas their inhibition alleviates nociception. In contrast, activation of GABAergic neurons produces analgesia, while their inhibition selectively restores social behavior. Distinct interneuron subtypes exhibit functional specialization: PV^+^ neurons regulate both pain sensitivity and social preference, whereas SST^+^ neurons selectively mediate social novelty processing. These interactions collectively indicate that chronic pain induces an E/I imbalance within ACC circuits, leading to dissociable regulation of pain and social behaviors.

Notably, optogenetic or chemogenetic activation of either PV^+^ or SST^+^ neurons failed to rescue pain-induced social deficits. Instead, inhibition of PV^+^ neurons selectively restored social preference, whereas inhibition of SST^+^ neurons selectively rescued social novelty (Figure 10). These findings indicate that ACC interneuron subtypes differentially regulate distinct stages of social behavior. This interpretation is consistent with our previous findings implicating PV⁺ neurons in social motivation and interaction 28. Protein tyrosine phosphatase 1B (PTP1B) is endogenously inhibited by LIM domain only protein 4 (LMO4), and loss of LMO4 is associated with autism-like phenotypes 29. PV^+^-specific deletion of LMO4 increases neuronal excitability and reduces social interaction, affects that are prevented by concurrent ablation of PTP1B, underscoring the importance of PV^+^ neurons in social behavior 12. In addition, PV^+^ neurons in the CA2 region contribute to social memory, and cortical SST^+^ neurons have also been implicated in social behavior 30,31. Our calcium imaging data further support this dissociation, showing that PV⁺ neurons encode social preference, whereas SST⁺ neurons encode social novelty. Together, the coordinated activity of these interneuron subtypes accounts for the dynamic modulation observed in the overall GABAergic population during social interaction.

VIP^+^ neurons, which preferentially target other inhibitory interneurons, have been implicated in social dominance and behavioral flexibility in the prefrontal cortex 10. However, in our study, ACC VIP^+^ neurons showed no significant modulation during pain or social behavior, and chemogenetic manipulation of this population had no detectable behavioral effects. This is consistent with previous imaging studies reporting heterogeneous and sparse VIP⁺ neuron responses during social stimuli, with no net change at the population level 32. These results suggest that while individual VIP⁺ neurons may encode specific social features, the overall ACC VIP⁺ population does not play a dominant role in pain or social behavior regulation.

Excitatory CaMKII^+^ neurons represent the principal output of the ACC and are tightly regulated by local inhibitory circuits. Our data demonstrate that inhibition of GABAergic neurons directly increases CaMKII⁺ neuronal activity in a dose-dependent manner, confirming functional inhibitory coupling and emphasizing the importance of E/I balance. Under physiological conditions, GABAergic neurons constrain CaMKII⁺ neuron activity, maintaining normal nociception and social behavior. In chronic pain, reduced inhibitory control leads to CaMKII⁺ neuron hyperactivity, driving both hyperalgesia and social deficits. Functionally, CaMKII⁺ neurons exhibited bidirectional and dissociable roles. Activation of CaMKII⁺ neurons selectively rescued social preference deficits but did not improve social novelty, whereas inhibition alleviated pain without restoring social behavior. These findings are consistent with previous studies showing that ACC CaMKII^+^ neuron activation improves social preference autism models 33,34. Together, our results indicate that CaMKII⁺ neurons participate in both pain and social circuits, but distinct activity states or downstream pathways likely underlie different behavioral outputs.

At the circuit level, our findings support a model in which pain-induced disruption of E/I balance in the ACC underlies social dysfunction. Decreased E/I ratio impaired social behavior in sham mice, whereas increasing E/I ratio ameliorated social deficits in pain-exposed mice. Conversely, lowering the E/I ratio alleviated pain. This bidirectional relationship is consistent with prior work linking altered E/I balance to social impairments in neurodevelopmental and neuropsychiatric disorders 35-37. Importantly, our study directly demonstrates functional interactions between GABAergic and CaMKII^+^ neurons in the ACC and establishes their causal roles in pain-social comorbidity.

Finally, beyond local circuit effects, altered ACC E/I balance may influence downstream brain networks involved in emotion, motivation, and social cognition. Thus, restoring E/I balance in the ACC may represent a promising strategy for treating social dysfunction associated with chronic pain. However, our findings also highlight a fundamental constraint: interventions targeting single neuronal populations are insufficient to simultaneously correct pain and social deficits. Further studies will need to develop circuit- and pathway-specific strategies capable of fine-tuning ACC network dynamics to achieve optimal therapeutic outcomes.

## Conclusion

In this study, we systematically delineated the functional contributions of distinct neuronal subtypes within the ACC to pain processing, social behavior, and pain-induced social deficits. Our findings demonstrate that CaMKII⁺ and PV⁺ neurons are key regulators of nociceptive processing, whereas CaMKII⁺, PV⁺, and SST⁺ neurons collectively govern social behavior. Notably, PV⁺ and SST⁺ neurons exert stage-specific control over social interactions, preferentially regulating social preference and social novelty, respectively. Despite these clearly defined roles, modulation of any single neuronal population was insufficient to simultaneously alleviate pain and restore social behavior. This dissociation supports the existence of a functional regulatory conflict within the ACC, in which neural mechanisms underlying pain relief and social recovery are partially segregated. Consequently, interventions targeting individual neuronal subtypes may improve one behavioral domain while leaving others unaffected or even disrupted. These findings underscore the complexity of ACC circuitry and suggest that additional neuronal subtypes, circuit motifs, or projection-specific pathways may contribute to the differential regulation of pain and social behavior. It is also likely that individual ACC neurons participate in multiple behavioral networks, such that altering their activity produces domain-specific benefits accompanied by unintended effects.

Together, our results highlight the necessity of developing circuit- and pathway-specific therapeutic strategies to decouple pain and social dysfunction. Continued investigation into the precise roles of ACC neuronal subtypes and their downstream connections will be essential for advancing targeted interventions for pain-associated social deficits and related neuropsychiatric conditions.

## Supplementary Material

Supplementary figures and data.

## Data Availability

The data that support the findings of this study are available from the corresponding author upon reasonable request.

## Abbreviations

ACC: anterior cingulate cortex
AICAR: 5-aminoimidazole-4-carboxamide ribonucleotide
AUC: area under the curve
CaMKII: calcium/calmodulin-dependent protein kinase II
CFA: complete Freund's adjuvant
ChR2: channelrhodopsin-2
CNO: clozapine N-oxide
E/I: excitatory/inhibitory
eNpHR: enhanced halorhodopsin
GABA: γ-aminobutyric acid
GAD: glutamate decarboxylase
GCaMP6s: genetically encoded calcium indicator 6s
hM3Dq: excitatory designer receptor exclusively activated by designer drugs
hM4Di: inhibitory designer receptor exclusively activated by designer drugs
PV: parvalbumin
SEM: standard error of the mean
SNI: spared nerve injury
SST: somatostatin
VIP: vasoactive intestinal peptide
WT: wild-type
