# Supplementary material for: Subtype-Specific Roles of Anterior Cingulate Cortex Neurons in Pain-Induced Social Deficits in Mice
Source: Theranostics. 2026 Feb 11;16(9):4619–40. doi: 10.7150/thno.129482 (PMC12964135; doi:10.7150/thno.129482)
Supplement: Supplementary file 1 — Supplementary figures and data. [file thnov16p4619s1.zip › Supplementary materials/Supplementary figure and legend.pdf]

Figure S1

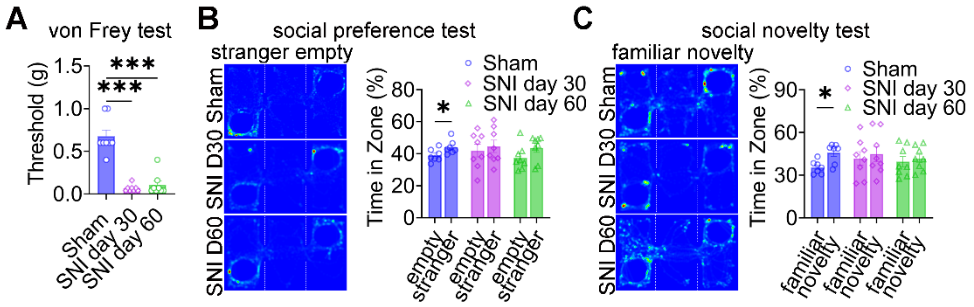

Figure S1. The neuropathic pain caused the mice to develop social deficits. (A) 30 and 60 days after SNI mice showed lower thresholds for mechanical pain (Sham: n=8; SNI day30: n=8; SNI day60: n=8; Mann Whitney test). (B) Representative heat maps and quantification of social time between Sham (n=8, Unpaired t test) and SNI day30 (n=8, Unpaired t test) and SNI day60 mice (n=8, Mann Whitney test) during the social preference test. (C) Representative heat maps and quantification of social time between Sham (n=7, Mann Whitney test) and SNI day30 (n=8, Unpaired t test) and SNI day60 mice (n=8, Unpaired t test) during the social novelty test.

Figure S2

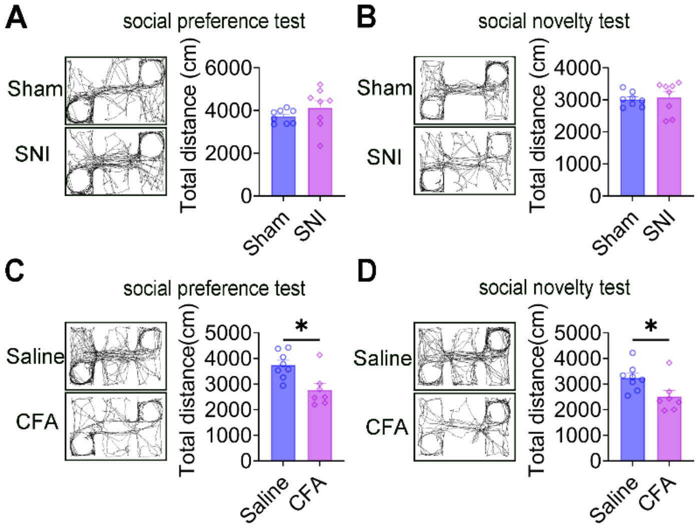

Figure S2. The effects of pain on the locomotor activity of mice. (A) Representative Motion trajectory diagram and quantification of total distance of motion between Sham and SNI mice (Sham: n=8, SNI: n=8, Unpaired t test) during the social preference test. (B) Representative Motion trajectory diagram and quantification of total distance of motion between Sham and SNI mice (Sham: n=8, SNI: n=8, Mann Whitney test) during the social novelty test. (C) Representative Motion trajectory diagram and quantification of total distance of motion between Saline and CFA mice (Saline: n=7, CFA: n=7, Unpaired t test) during the social preference test. (D) Representative Motion trajectory diagram and quantification of total distance of motion between Sham and CFA mice (Saline: n=7, CFA: n=7, Mann Whitney test) during the social novelty test.

Figure S3

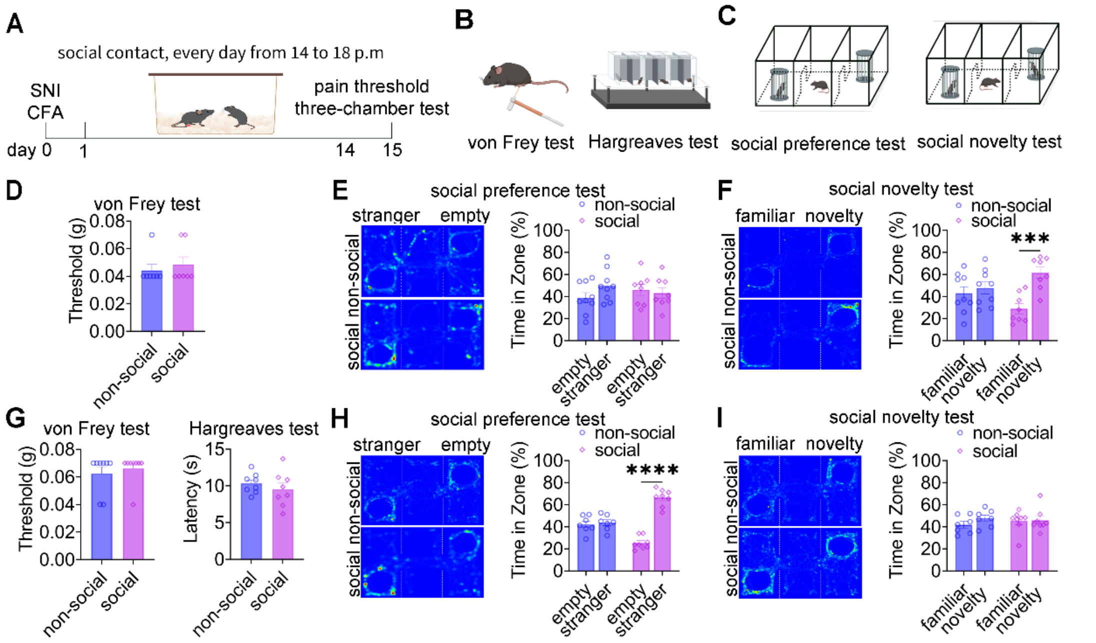

Figure S3. The effects of passive social interaction on pain and social behavior in mice. (A) Experimental flow chart. (B-C) Experimental model diagram showing mice von Frey test, Hargreaves test and three-chamber test. (D) There was no significant difference in mechanical pain between non-social and social group. (non-social: n=7, social n=7, P=0.0002, Mann Whitney test). (E) Representative heat maps and quantification of social time between non-social (n=9, Unpaired t test) and social group (n=8, Unpaired t test) during the social preference test. (F) Representative heat maps and quantification of social time between non-social (n=9, Unpaired t test) and social group (n=8, Unpaired t test) during the social novelty test. (G) There was no significant difference in mechanical pain (non-social: n=8, social n=8, Mann Whitney test) and thermal pain between non-social and social group (non-social: n=8, social: n=8, Unpaired t test). (H) Representative heat maps and quantification of social time between non-social (n=7, Unpaired t test) and social group (n=8, Unpaired t test) during the social preference test. (I) Representative heat maps and quantification of social time between non-social (n=7, Unpaired t test) and social group (n=8, Mann Whitney test) during the social novelty test.

Figure S4

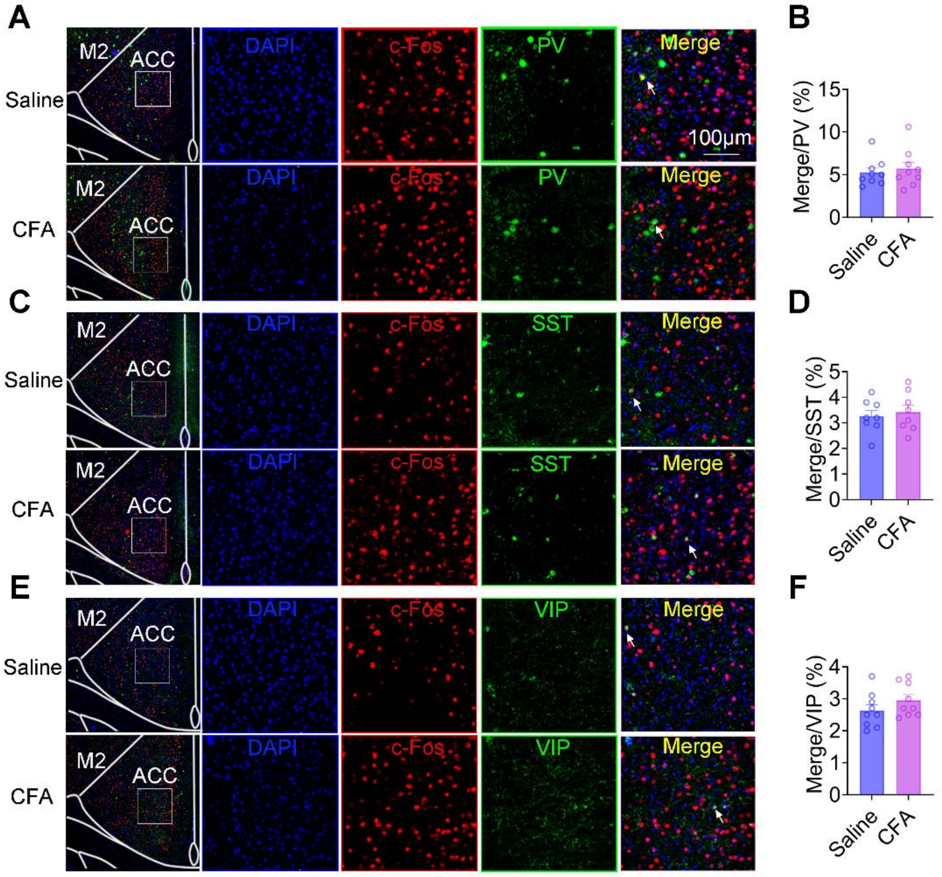

Figure S4. c-Fos activation in different GABAergic neuron subtypes in the ACC of CFA mice. (A) Typical images of PV neurons and c-Fos immunofluorescence staining in ACC of Saline and CFA mice (bar=100μm). (B) Statistical diagram of c-Fos activation percentage in PV neurons in ACC of Saline and CFA mice (Saline: n=9, CFA: n=9, Unpaired t test). (C) Typical images of SST neurons and c-Fos immunofluorescence staining in ACC of Saline and CFA mice (bar=100μm). (D) Statistical diagram of c-Fos activation percentage in SST neurons in ACC of Saline and CFA mice (Saline: n=9, CFA: n=9, Unpaired t test). (E) Typical images of VIP neurons and c-Fos immunofluorescence staining in ACC of Saline and CFA mice (bar=100μm). (F) Statistical diagram of c-Fos activation percentage in VIP neurons in ACC of Saline and CFA mice (Saline: n=9, CFA: n=9, Unpaired t test).

Figure S5

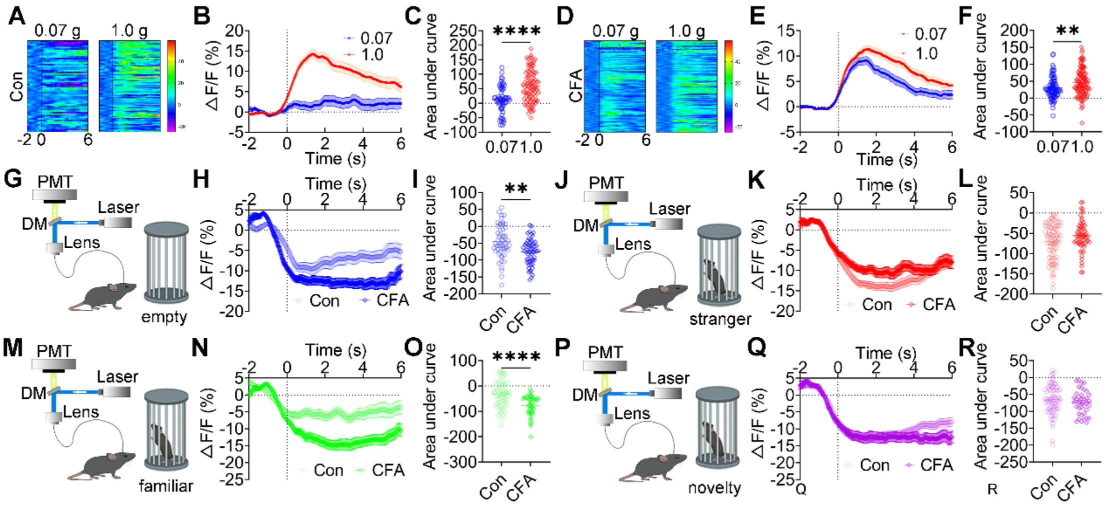

Figure S5. The effects of mechanical nociceptive stimulation and social interaction on the activity of ACC GABAergic neurons in CFA mice. (A) Heat map illustrating the calcium response of ACC GABAergic neurons in Sham mice during von Frey stimulation. (B, C) Peri-event plots and statistical graphs of averaged calcium signals (0.07 g: n=59 trials from six mice, 1.0 g: n=92 trials from six mice, Unpaired t test). (D) Heat map illustrating the calcium response of ACC GABAergic neurons in CFA mice during von Frey stimulation. (E, F) Peri-event plots and statistical graphs of averaged calcium signals (0.07 g: n=85 trials from six mice, 1.0 g: n=111 trials from six mice, Mann Whitney test). (G) Pattern plot of calcium signals when mouse approaches empty cage. (H, I) Peri-event plots and statistical graphs of averaged calcium signals (Con: n=52 trials from six mice, CFA: n=56 trials from six mice, Unpaired t test). (J) Pattern plot of calcium signals when mouse approaches stranger mouse. (K, L) Peri-event plots and statistical graphs of averaged calcium signals (Con: n=85 trials from six mice, CFA: n=60 trials from six mice, Mann Whitney test). (M) Pattern plot of calcium signals when mouse approaches familiar mouse. (N, O) Peri-event plots and statistical graphs of averaged calcium signals (Con: n=53trials from six mice, CFA: n=42 trials from six mice, Mann Whitney test). (P) Pattern plot of calcium signals when mouse approaches novelty mouse. (Q, R) Peri-event plots and statistical graphs of averaged calcium signals (Con: n=72 trials from six mice, CFA: n=38 trials from six mice, Unpaired t test).

Figure S6

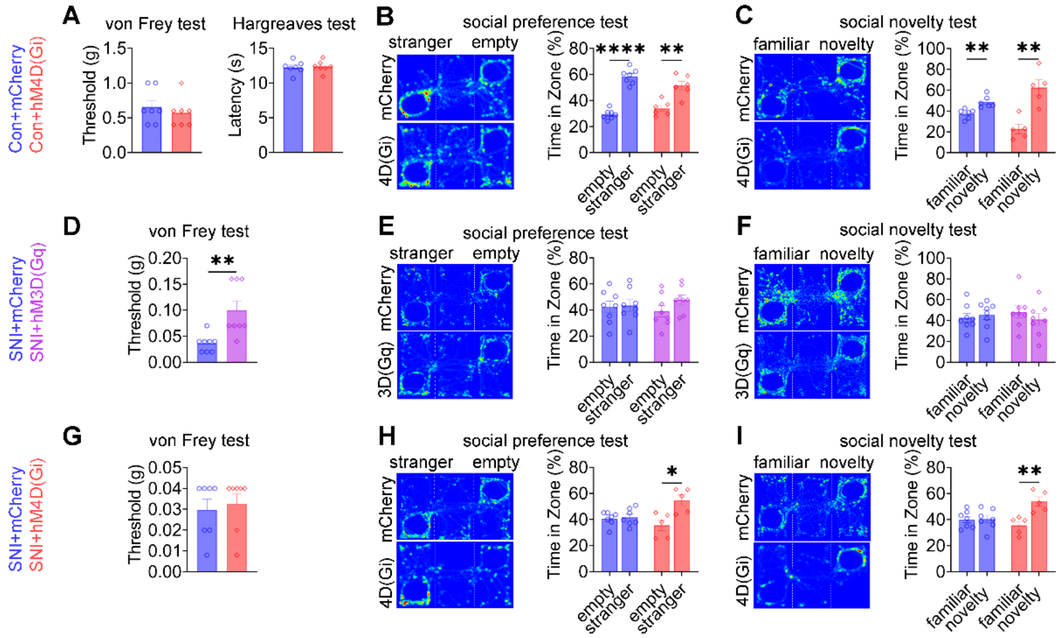

Figure S6 The effects of chemogenetic manipulation of ACC GABAergic neurons on pain and social behavior in mice. (A) There was no significant difference in mechanical pain (mCherry: n=7, hM4D(Gi): n=7, Mann Whitney test) and thermal pain (mCherry: n=7, hM4D(Gi): n=7, Unpaired t test) between mCherry and hM4D(Gi) groups. (B) Representative heat maps and quantification of social time between mCherry (n=7; Unpaired t test) and hM4D(Gi) mice (n=6; Unpaired t test) during the social preference test. (C) Representative heat maps and quantification of social time between mCherry (n=6; Unpaired t test) and hM4D(Gi) mice (n=5; Unpaired t test) during the social novelty test. (D) Mechanical pain was relieved in the hM3D(Gq) compared with mCherry (mCherry: n=8, hM3D(Gq): n=8; Mann Whitney test). (E) Representative heat maps and quantification of social time between mCherry (n=8; Unpaired t test) and hM3D(Gq) mice (n=8; Unpaired t test) during the social preference test. (F) Representative heat maps and quantification of social time between mCherry (n=8, Unpaired t test) and hM3D(Gq) mice (n=8, Unpaired t test) during the social novelty test. (G) There was no significant difference in mechanical pain between mCherry and hM4D(Gi) groups (mCherry: n=7, hM4D(Gi): n=7, Mann Whitney test). (H) Representative heat maps and quantification of social time between mCherry (n=7; Unpaired t test) and hM4D(Gi) mice (n=5; Unpaired t test) during the social preference test. (I) Representative heat maps and quantification of social time between mCherry (n=7; Unpaired t test) and hM4D(Gi) mice (n=5; Unpaired t test) during the social novelty test.

Figure S7

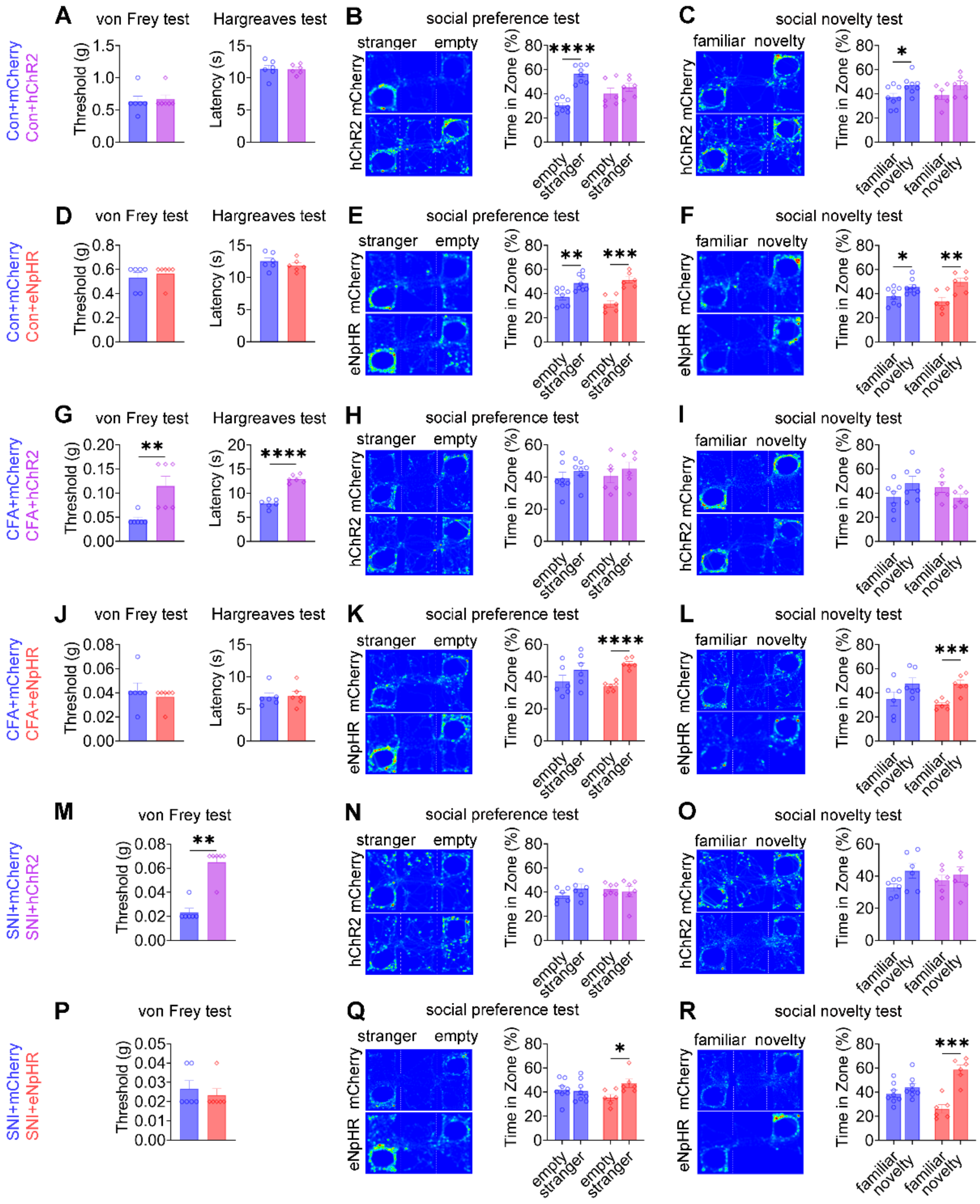

Figure S7 The effects of optogenetic manipulation of ACC GABAergic neurons on pain and social behavior in mice. (A) There was no significant difference in mechanical pain (mCherry: n=6, hChR2: n=6; Mann Whitney test) and thermal pain (mCherry: n=6, hChR2: n=6; Unpaired t test) between mCherry and hChR2 groups. (B) Representative heat maps and quantification of social time between mCherry (n=8; Unpaired t test) and hChR2 mice (n=6; Unpaired t test) during the social preference test. (C) Representative heat maps and quantification of social time between mCherry (n=8; Unpaired t test) and hChR2 mice (n=6; Unpaired t test) during the social novelty test. (D) There was no significant difference in mechanical pain (mCherry: n=6, eNpHR: n=6, Mann Whitney test) and thermal pain (mCherry: n=6, eNpHR: n=6, Unpaired t test)

between mCherry and eNpHR groups. (E) Representative heat maps and quantification of social time between mCherry (n=8, Unpaired t test) and eNpHR mice (n=6; Unpaired t test) during the social preference test. (F) Representative heat maps and quantification of social time between mCherry (n=8, Unpaired t test) and eNpHR mice (n=6; Unpaired t test) during the social novelty test. (G) Both mechanical pain (mCherry: n=6, hChR2: n=6, Mann Whitney test) and thermal pain (mCherry: n=6, hChR2: n=6, Unpaired t test) were relieved in the hChR2 compared with mCherry mice. (H) Representative heat maps and quantification of social time between mCherry (n=7; Unpaired t test) and hChR2 mice (n=6; Unpaired t test) during the social preference test. (I) Representative heat maps and quantification of social time between mCherry (n=7; Unpaired t test) and hChR2 mice (n=6; Unpaired t test) during the social novelty test. (J) There was no significant difference in mechanical pain (mCherry: n=6, eNpHR: n=6, Mann Whitney test) and thermal pain (mCherry: n=6, eNpHR: n=6, Mann Whitney test) between mCherry and eNpHR groups. (K) Representative heat maps and quantification of social time between mCherry (n=6; Unpaired t test) and eNpHR mice (n=6; Unpaired t test) during the social preference test. (L) Representative heat maps and quantification of social time between mCherry (n=6; Unpaired t test) and eNpHR mice (n=6; Unpaired t test) during the social novelty test. (M) Mechanical pain was relieved in the hChR2 compared with mCherry mice (mCherry: n=6, hChR2: n=6, Mann Whitney test). (N) Representative heat maps and quantification of social time between mCherry (n=6; Unpaired t test) and hChR2 mice (n=6; Unpaired t test) during the social preference test. (O) Representative heat maps and quantification of social time between mCherry (n=6; Unpaired t test) and hChR2 mice (n=6; Unpaired t test) during the social novelty test. (P) There was no significant difference in mechanical pain (mCherry: n=6, eNpHR: n=6, Mann Whitney test) between mCherry and eNpHR groups. (Q) Representative heat maps and quantification of social time between mCherry (n=8; Unpaired t test) and eNpHR mice (n=6; Mann Whitney test) during the social preference test. (R) Representative heat maps and quantification of social time between mCherry (n=8; Unpaired t test) and eNpHR mice (n=6; Mann Whitney test) during the social novelty test.

Figure S8

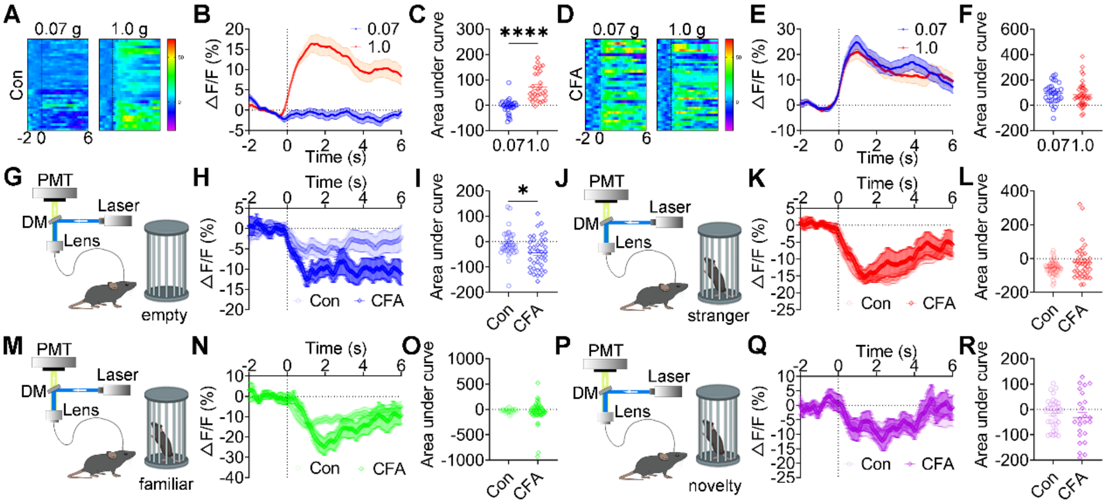

Figure S8. The effects of mechanical nociceptive stimulation and social interaction on the activity of ACC PV neurons in CFA mice. (A) Heat map illustrating the calcium response of ACC PV neurons in Sham mice during von Frey stimulation. (B, C) Peri-event plots and statistical graphs of averaged calcium signals (0.07 g: n=31 trials from six mice, 1.0 g: n=30 trials from six mice, Mann Whitney test). (D) Heat map illustrating the calcium response of ACC PV neurons in CFA mice during von Frey stimulation. (E, F) Peri-event plots and statistical graphs of averaged calcium signals (0.07 g: n=33 trials from six mice, 1.0 g: n=40 trials from six mice, Mann Whitney test). (G) Pattern plot of calcium signals when mouse approaches empty cage. (H, I) Peri-event plots and statistical graphs of averaged calcium signals (Con: n=35 trials from six mice, CFA: n=38 trials from six mice, Mann Whitney test). (J) Pattern plot of calcium signals when mouse approaches stranger mouse. (K, L) Peri-event plots and statistical graphs of averaged calcium signals (Con: n=48 trials from six mice, CFA: n=38 trials from six mice, Mann Whitney test). (M) Pattern plot of calcium signals when mouse approaches familiar mouse. (N, O) Peri-event plots and statistical graphs of averaged calcium signals (Con: n=28trials from six mice, CFA: n=49 trials from six mice, Mann Whitney test). (P) Pattern plot of calcium signals when mouse approaches novelty mouse. (Q, R) Peri-event plots and statistical graphs of averaged calcium signals (Con: n=32 trials from six mice, CFA: n=24 trials from six mice, Unpaired t test).

Figure S9

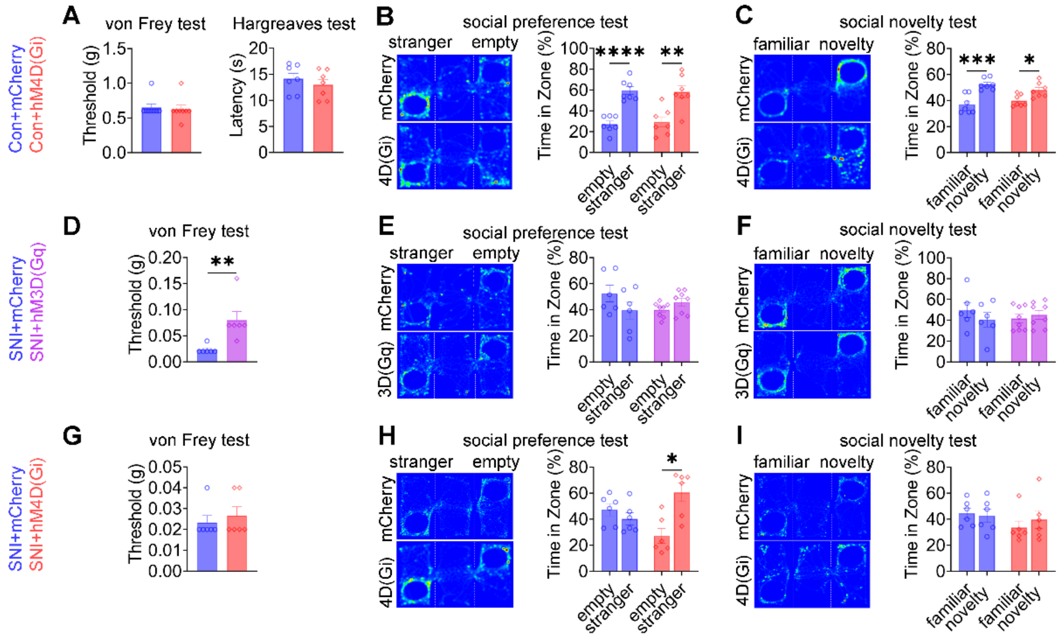

Figure S9. The effects of chemogenetic manipulation of ACC PV neurons on pain and social behavior in mice. (A) There was no significant difference in mechanical pain (mCherry: n=8, hM4D(Gi): n=8, Mann Whitney test) and thermal pain (mCherry: n=7, hM4D(Gi): n=7, Unpaired t test) between mCherry and hM4D(Gi) groups. (B) Representative heat maps and quantification of social time between mCherry (n=7; Unpaired t test) and hM4D(Gi) mice (n=7, Unpaired t test) during the social preference test. (C) Representative heat maps and quantification of social time between mCherry (n=7; Unpaired t test) and hM4D(Gi) mice (n=7, Unpaired t test) during the social novelty test. (D) Mechanical pain were relieved in the hM3D(Gq) compared with mCherry (mCherry: n=6, hM3D(Gq): n=6, Mann Whitney test). (E) Representative heat maps and quantification of social time between mCherry (n=6; Unpaired t test) and hM3D(Gq) mice (n=8; Unpaired t test) during the social preference test. (F) Representative heat maps and quantification of social time between mCherry (n=6; Unpaired t test) and hM3D(Gq) mice (n=8; Unpaired t test) during the social novelty test. (G) There was no significant difference in mechanical pain between mCherry and hM4D(Gi) groups (mCherry: n=6, hM4D(Gi): n=6, Mann Whitney test). (H) Representative heat maps and quantification of social time between mCherry (n=6; Unpaired t test) and hM4D(Gi) mice (n=6, Mann Whitney test) during the social preference test. (I) Representative heat maps and quantification of social time between mCherry (n=6; Unpaired t test) and hM4D(Gi) mice (n=6, Mann Whitney test) during the social novelty test.

Figure S10

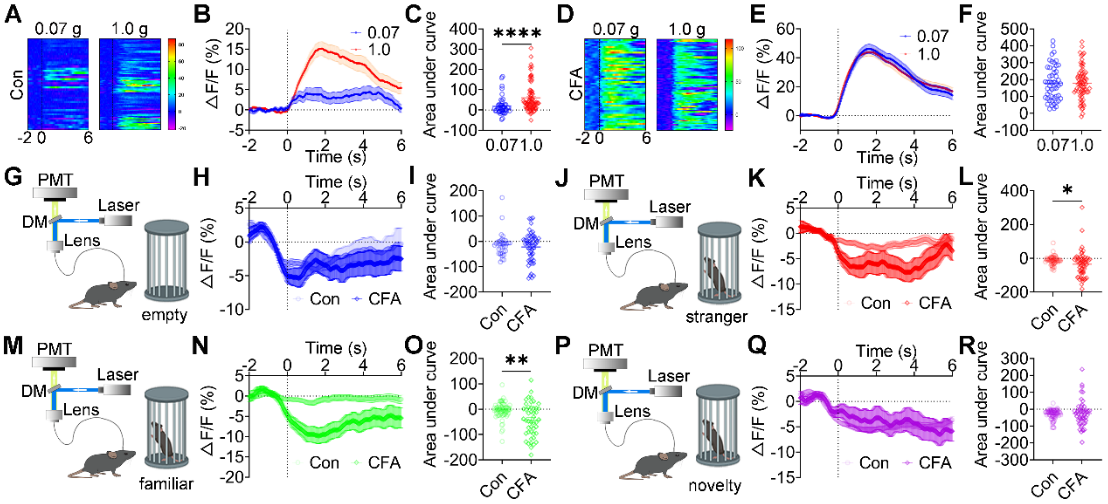

Figure S10. The effects of mechanical nociceptive stimulation and social interaction on the activity of ACC SST neurons in CFA mice. (A) Heat map illustrating the calcium response of ACC SST neurons in Sham mice during von Frey stimulation. (B, C) Peri-event plots and statistical graphs of averaged calcium signals (0.07 g: n=44 trials from six mice, 1.0 g: n=77 trials from six mice, Mann Whitney test). (D) Heat map illustrating the calcium response of ACC SST neurons in CFA mice during von Frey stimulation. (E, F) Peri-event plots and statistical graphs of averaged calcium signals (0.07 g: n=51 trials from six mice, 1.0 g: n=70 trials from six mice, Unpaired t test). (G) Pattern plot of calcium signals when mouse approaches empty cage. (H, I) Peri-event plots and statistical graphs of averaged calcium signals(Con: n=35 trials from six mice，CFA: n=52 trials from six mice, Mann Whitney test). (J) Pattern plot of calcium signals when mouse approaches stranger mouse. (K, L) Peri-event plots and statistical graphs of averaged calcium signals (Con: n=61 trials from six mice, CFA: n=56 trials from six mice, Mann Whitney test). (M) Pattern plot of calcium signals when mouse approaches familiar mouse. (N, O) Peri-event plots and statistical graphs of averaged calcium signals (Con: n=70 trials from six mice, CFA: n=46 trials from six mice, Mann Whitney test). (P) Pattern plot of calcium signals when mouse approaches novelty mouse. (Q, R) Peri-event plots and statistical graphs of averaged calcium signals (Con: n=86 trials from six mice, CFA: n=51 trials from six mice, Mann Whitney test).

Figure S11

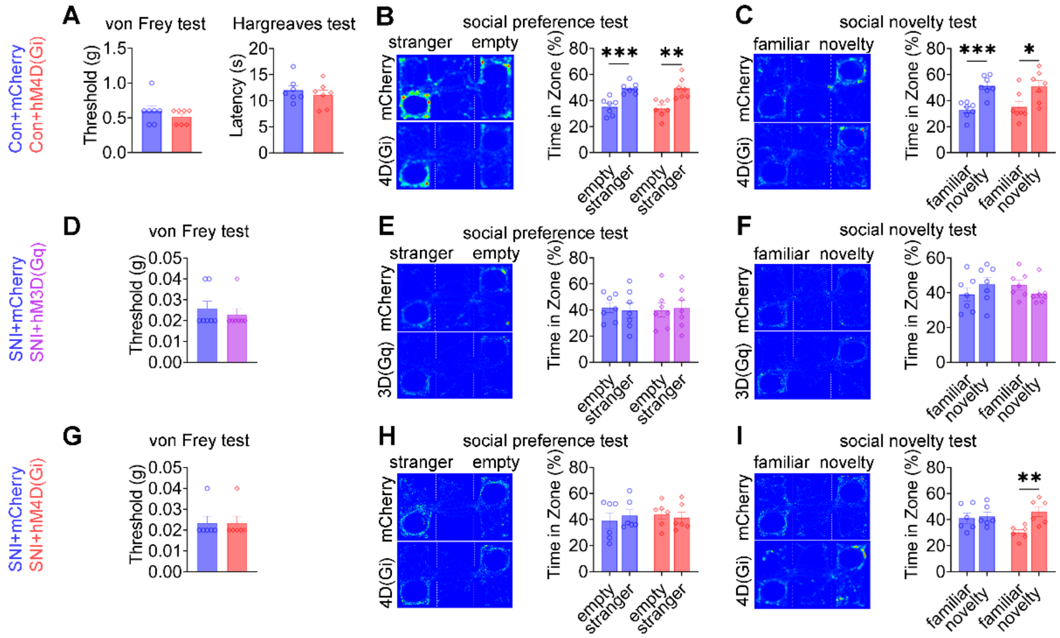

Figure S11. The effects of chemogenetic manipulation of ACC SST neurons on pain and social behavior in mice. (A) There was no significant difference in mechanical pain (mCherry: n=7, hM4D(Gi): n=7, Mann Whitney test) and thermal pain (mCherry: n=7, hM4D(Gi): n=7, Unpaired t test) between mCherry and hM4D(Gi) groups. (B) Representative heat maps and quantification of social time between mCherry (n=7, Unpaired t test) and hM4D(Gi) mice (n=7, Unpaired t test) during the social preference test. (C) Representative heat maps and quantification of social time between mCherry (n=7, Unpaired t test) and hM4D(Gi) mice (n=7, Unpaired t test) during the social novelty test. (D) There was no significant difference in mechanical pain between mCherry and hM3D(Gq) groups. (mCherry: n=7, hM3D(Gq): n=7, Mann Whitney test). (E) Representative heat maps and quantification of social time between mCherry (n=7, Unpaired t test) and hM3D(Gq) mice (n=7; Unpaired t test) during the social preference test. (F) Representative heat maps and quantification of social time between mCherry (n=7, Unpaired t test) and hM3D(Gq) mice (n=7; Mann Whitney test) during the social novelty test. (G) There was no significant difference in mechanical pain between mCherry and hM4D(Gi) groups (mCherry: n=6, hM4D(Gi): n=6, Mann Whitney test). (H) Representative heat maps and quantification of social time between mCherry (n=6, Mann Whitney test) and hM4D(Gi) mice (n=6, Unpaired t test) during the social preference test. (I) Representative heat maps and quantification of social time between mCherry (n=6, Unpaired t test) and hM4D(Gi) mice (n=6, Unpaired t test) during the social novelty test.

Figure S12

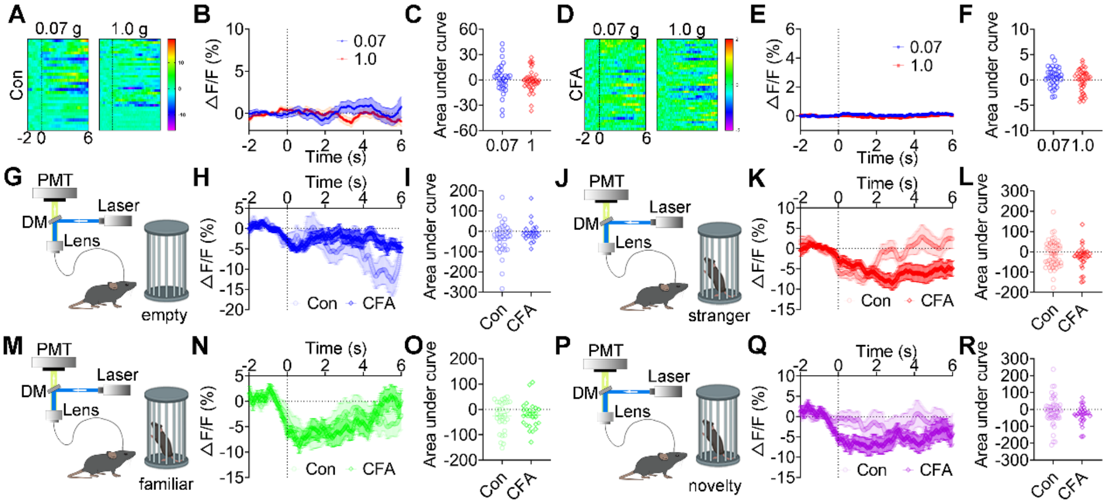

Figure S12. The effects of mechanical nociceptive stimulation and social interaction on the activity of ACC VIP neurons in CFA mice. (A) Heat map illustrating the calcium response of ACC VIP neurons in Sham mice during von Frey stimulation. (B, C) Peri-event plots and statistical graphs of averaged calcium signals (0.07 g: n=30 trials from four mice, 1.0 g: n=38 trials from four mice, Mann Whitney test). (D) Heat map illustrating the calcium response of ACC VIP neurons in CFA mice during von Frey stimulation. (E, F) Peri-event plots and statistical graphs of averaged calcium signals (0.07 g: n=33 trials from four mice, 1.0 g: n=39 trials from four mice, Unpaired t test). (G) Pattern plot of calcium signals when mouse approaches empty cage. (H, I) Peri-event plots and statistical graphs of averaged calcium signals (Con: n=33 trials from four mice, CFA: n=20 trials from four mice, Mann Whitney test). (J)Pattern plot of calcium signals when mouse approaches stranger mouse. (K, L) Peri-event plots and statistical graphs of averaged calcium signals (Con: n=57 trials from four mice, CFA: n=31 trials from four mice, Mann Whitney test). (M) Pattern plot of calcium signals when mouse approaches familiar mouse. (N, O) Peri-event plots and statistical graphs of averaged calcium signals (Con: n=30 trials from four mice, CFA: n=26 trials from four mice, Mann Whitney test). (P) Pattern plot of calcium signals when mouse approaches novelty mouse. (Q, R) Peri-event plots and statistical graphs of averaged calcium signals (Con: n=51 trials from four mice, CFA: n=28 trials from four mice, Unpaired t test).

Figure S13

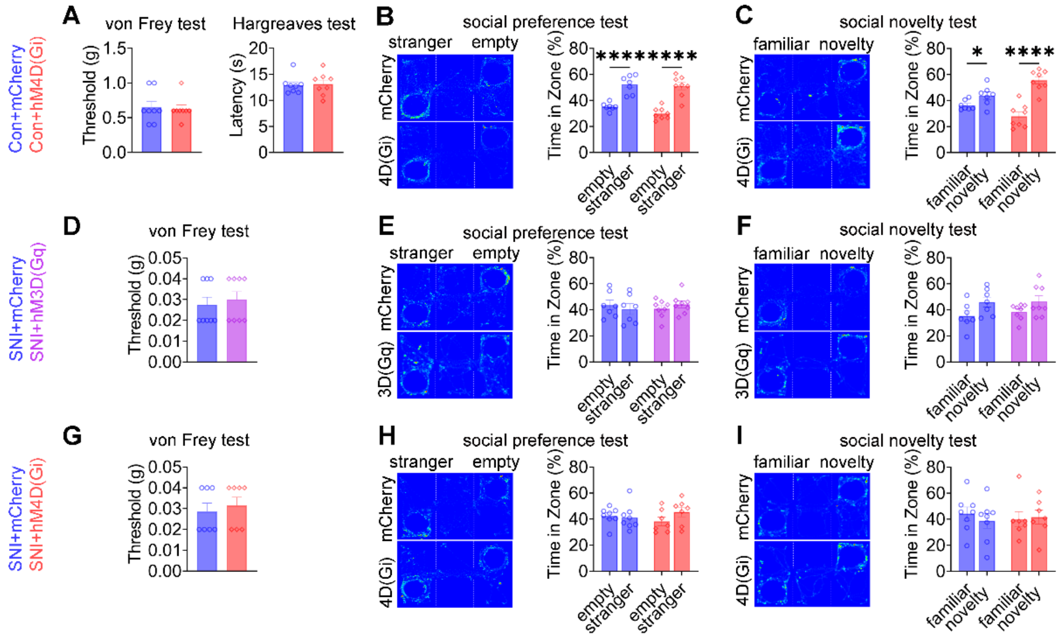

Figure S13. The effects of chemogenetic manipulation of ACC VIP neurons on pain and social behavior in mice. (A) There was no significant difference in mechanical pain (mCherry: n=8, hM4D(Gi): n=8, Mann Whitney test) and thermal pain (mCherry: n=8, hM4D(Gi): n=8, Mann Whitney test) between mCherry and hM4D(Gi) groups. (B) Representative heat maps and quantification of social time between mCherry (n=7, Unpaired t test) and hM4D(Gi) mice (n=8, Unpaired t test) during the social preference test. (C) Representative heat maps and quantification of social time between mCherry (n=7, Unpaired t test) and hM4D(Gi) mice (n=8, Unpaired t test) during the social novelty test. (D) There was no significant difference in mechanical pain between mCherry and hM3D(Gq) groups (mCherry: n=8, hM3D(Gq): n=8, Mann Whitney test). (E) Representative heat maps and quantification of social time between mCherry (n=7, Unpaired t test) and hM3D(Gq) mice (n=8, Unpaired t test) during the social preference test. (F) Representative heat maps and quantification of social time between mCherry (n=7, Unpaired t test) and hM3D(Gq) mice (n=8, Unpaired t test) during the social novelty test. (G) There was no significant difference in mechanical pain between mCherry and hM4D(Gi) groups (mCherry: n=7, hM4D(Gi): n=7, Mann Whitney test). (H) Representative heat maps and quantification of social time between mCherry (n=8, Unpaired t test) and hM4D(Gi) mice (n=7, Unpaired t test) during the social preference test. (I) Representative heat maps and quantification of social time between mCherry (n=8, Unpaired t test) and hM4D(Gi) mice (n=7, Mann Whitney test) during the social novelty test.

Figure S14

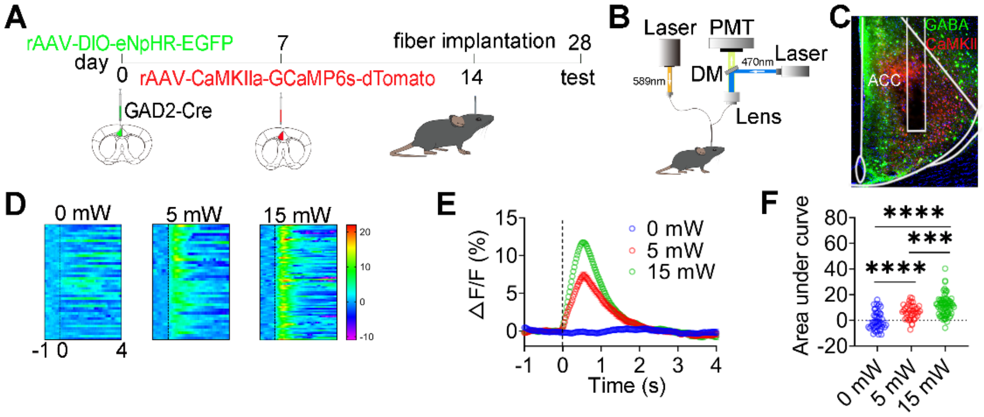

Figure S14. Effects of inhibiting ACC GABA on CaMKII neuronal activity. (A) Experimental workflow diagram. (B) Schematic diagram of optogenetics combined with calcium imaging. (C) Representative image of viral expression at injection site. (D) Representative heatmaps of calcium imaging under different light intensity patterns. (E) Peri-event plots of averaged calcium signals. (F) Statistical graphs of area under the curve for averaged calcium signals.

Figure S15

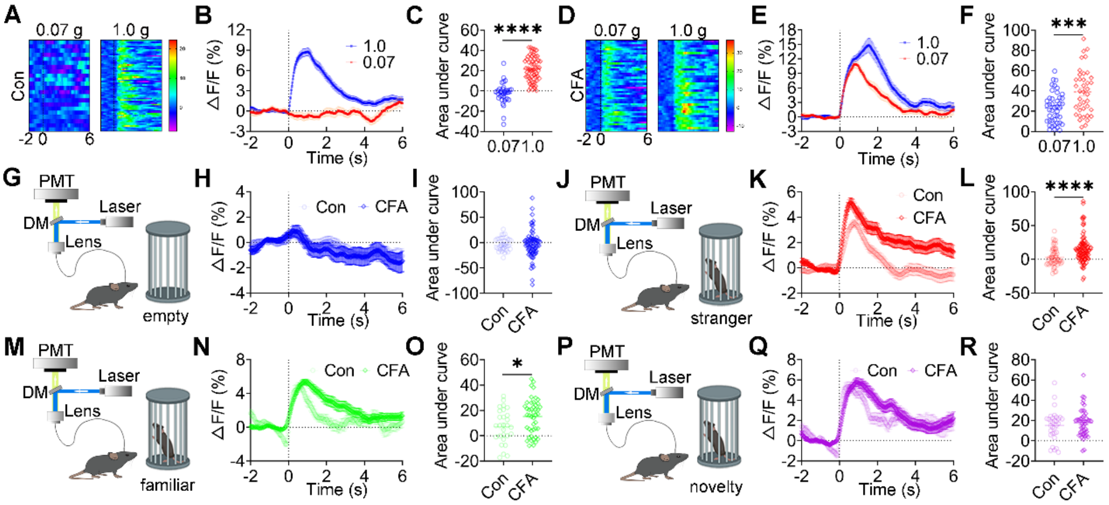

Figure S15. The effects of mechanical nociceptive stimulation and social interaction on the activity of ACC CaMKII neurons in CFA mice. (A) Heat map illustrating the calcium response of ACC CaMKII neurons in Sham mice during von Frey stimulation. (B, C) Peri-event plots and statistical graphs of averaged calcium signals (0.07 g: n=35 trials from five mice, 1.0 g: n=61 trials from five mice, Mann Whitney test). (D) Heat map illustrating the calcium response of ACC CaMKII neurons in CFA mice during von Frey stimulation. (E, F) Peri-event plots and statistical graphs of averaged calcium signals (0.07 g: n=44 trials from five mice, 1.0 g: n=39 trials from five mice, Unpaired t test). (G) Pattern plot of calcium signals when mouse approaches empty cage. (H, I) Peri-event plots and statistical graphs of averaged calcium signals (Con: n=37 trials from five mice, CFA: n=78 trials from five mice, Mann Whitney test). (J) Pattern plot of calcium signals when mouse approaches stranger mouse. (K, L) Peri-event plots and statistical graphs of averaged calcium signals (Con: n=55 trials from five mice, CFA: n=98 trials from five mice, Mann Whitney test). (M) Pattern plot of calcium signals when mouse approaches familiar mouse. (N, O) Peri-event plots and statistical graphs of averaged calcium signals (Con: n=28 trials from five mice, CFA: n=45 trials from five mice, Mann Whitney test). (P) Pattern plot of calcium signals when mouse approaches novelty mouse. (Q, R) Peri-event plots and statistical graphs of averaged calcium signals (Con: n=32 trials from five mice, CFA: n=45 trials from five mice, Unpaired t test).

Figure S16

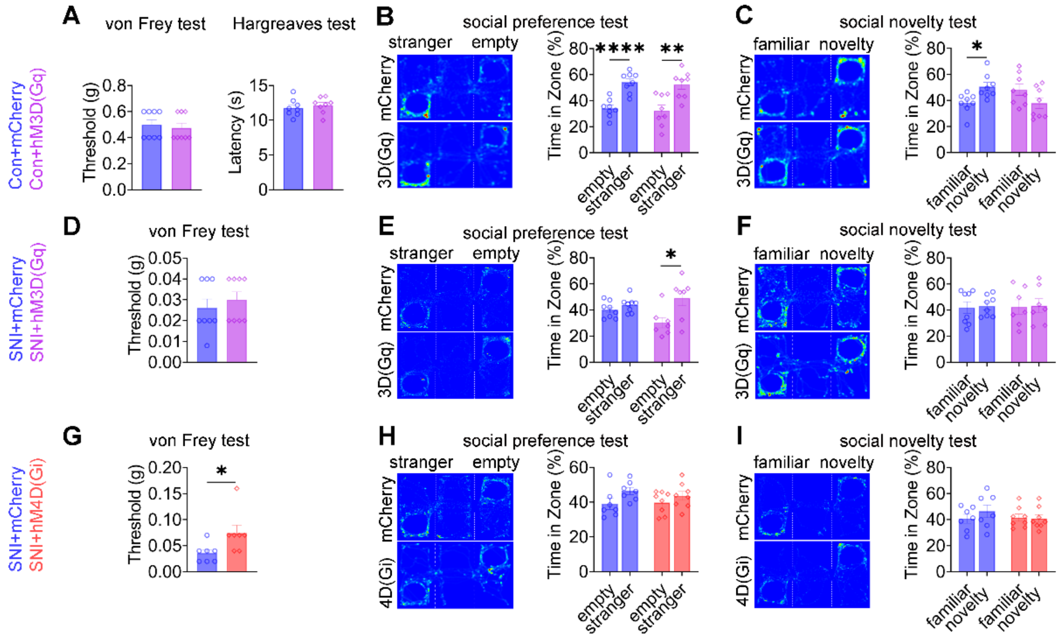

Figure S16. The effects of chemogenetic manipulation of ACC CaMKII neurons on pain and social behavior in mice. (A) There was no significant difference in mechanical pain (mCherry: n=8, hM3D(Gq): n=8, Mann Whitney test) and thermal pain (mCherry: n=8, hM3D(Gq): n=8, Unpaired t test) between mCherry and hM3D(Gq). (B) Representative heat maps and quantification of social time between mCherry (n=8, Unpaired t test) and hM3D(Gq) mice (n=8, Unpaired t test) during the social preference test. (C) Representative heat maps and quantification of social time between mCherry (n=8; Unpaired t test) and hM3D(Gq) mice (n=8; Unpaired t test) during the social novelty test. (D) There was no significant difference in mechanical pain between mCherry and hM3D(Gq) groups (mCherry: n=8, hM3D(Gq): n=8, Mann Whitney test). (E) Representative heat maps and quantification of social time between mCherry (n=8, Unpaired t test) and hM3D(Gq) mice (n=7, Unpaired t test) during the social preference test. (F) Representative heat maps and quantification of social time between mCherry (n=8, Mann Whitney test) and hM3D(Gq) mice (n=7, Unpaired t test) during the social novelty test. (G) Mechanical pain were relieved in the hM4D(Gi) compared with mCherry (mCherry: n=7; hM4D(Gi): n=7, Mann Whitney test). (H) Representative heat maps and quantification of social time between mCherry (n=7, Unpaired t test) and hM4D(Gi) mice (n=8, Unpaired t test) during the social preference test. (I) Representative heat maps and quantification of social time between mCherry (n=7, Unpaired t test) and hM4D(Gi) mice (n=8, Unpaired t test) during the social novelty test.

Figure S17

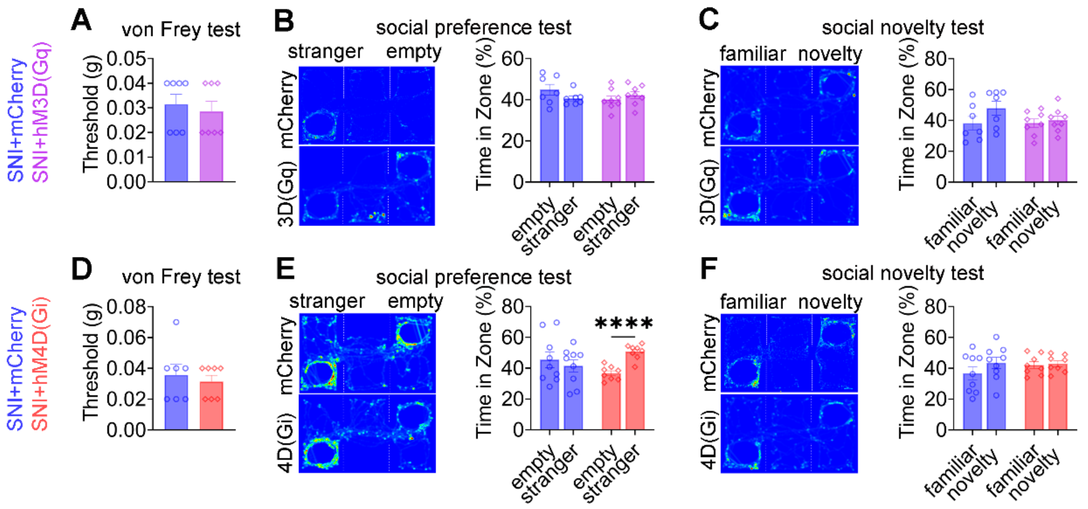

Figure S17. The effects of chemogenetic manipulation of ACC neurons on pain and social behavior in mice. (A) There was no significant difference in mechanical pain between mCherry and hM3D(Gq) groups. (mCherry: n=7, hM3D(Gq): n=7, Mann Whitney test). (B) Representative heat maps and quantification of social time between mCherry (n=7, Unpaired t test) and hM3D(Gq) mice (n=8, Unpaired t test) during the social preference test. (C) Representative heat maps and quantification of social time between mCherry (n=7, Mann Whitney test) and hM3D(Gq) mice (n=8, Unpaired t test) during the social novelty test. (D) There was no significant difference in mechanical pain between mCherry and hM4D(Gi) mice (mCherry: n=7, hM4D(Gi): n=7, Mann Whitney test). (E) Representative heat maps and quantification of social time between mCherry (n=9, Unpaired t test) and hM4D(Gi) mice (n=8, Unpaired t test) during the social preference test. (F) Representative heat maps and quantification of social time between mCherry (n=9, Unpaired t test) and hM4D(Gi) mice (n=8, Unpaired t test) during the social novelty test.
